# Health effects of protein intake in healthy adults: a systematic literature review

**DOI:** 10.3402/fnr.v57i0.21245

**Published:** 2013-07-30

**Authors:** Agnes N. Pedersen, Jens Kondrup, Elisabet Børsheim

**Affiliations:** 1 DTU Food, National Food Institute, Lyngby, Denmark; 2 Clinical Nutrition Unit, Rigshospitalet University Hospital, Copenhagen, Denmark; 3 Department of Surgery, The University of Texas Medical Branch, Galveston, TX, USA

**Keywords:** protein requirement, nitrogen balance, animal protein, vegetable protein, mortality, chronic disease, Nordic nutrition recommendations

## Abstract

The purpose of this systematic review is to assess the evidence behind the dietary requirement of protein and to assess the health effects of varying protein intake in healthy adults. The literature search covered the years 2000–2011. Prospective cohort, case-control, and intervention studies were included. Out of a total of 5,718 abstracts, 412 full papers were identified as potentially relevant, and after careful scrutiny, 64 papers were quality graded as A (highest), B, or C. The grade of evidence was classified as *convincing, probable, suggestive* or *inconclusive*. The evidence is assessed as: *probable* for an estimated average requirement of 0.66 g good-quality protein/kg body weight (BW)/day based on nitrogen balance studies, *suggestive* for a relationship between increased all-cause mortality risk and long-term low-carbohydrate–high-protein (LCHP) diets; but *inconclusive* for a relationship between all-cause mortality risk and protein intake *per se*; *suggestive* for an inverse relationship between cardiovascular mortality and vegetable protein intake; *inconclusive* for relationships between cancer mortality and cancer diseases, respectively, and protein intake; *inconclusive* for a relationship between cardiovascular diseases and total protein intake; *suggestive* for an inverse relationship between blood pressure (BP) and vegetable protein; *probable* to *convincing* for an inverse relationship between soya protein intake and LDL cholesterol; *inconclusive* for a relationship between protein intake and bone health, energy intake, BW control, body composition, renal function, and risk of kidney stones, respectively; *suggestive* for a relationship between increased risk of type 2 diabetes (T2D) and long-term LCHP-high-fat diets; *inconclusive* for impact of physical training on protein requirement; and *suggestive* for effect of physical training on whole-body protein retention. In conclusion, the evidence is assessed as *probable* regarding the estimated requirement based on nitrogen balance studies, and *suggestive* to *inconclusive* for protein intake and mortality and morbidity. Vegetable protein intake was associated with decreased risk in many studies. Potentially adverse effects of a protein intake exceeding 20–23 E% remain to be investigated.

This literature review is part of the fifth version of the Nordic Nutrition Recommendations (NNR5) project with the aim of reviewing and updating the scientific basis of the fourth edition of the NNR issued in 2004 (1). The NNR5 project is mainly focused on a revision of those areas in which new scientific knowledge has emerged since the fourth edition, with special relevance for the Nordic setting. A number of systematic literature reviews form the basis for establishment of dietary reference values in NNR5. The present expert group was established to systematically review studies regarding nitrogen balance (N-balance) and protein quantity and quality associated with health outcomes.

In 2002, the IoM published the US dietary reference values for protein (2) that was mainly based on a meta-analysis of N-balance studies by Rand et al. (3) to estimate protein requirement. This meta-analysis was also taken into consideration in the NNR in 2004 (NNR4) protein requirement assessment, while the recommendation was expressed as the energy percentage (E%) from protein, which also allowed for the macronutrient intake distribution and the Nordic dietary habits. The Nordic-recommended protein intake of 10–20 E% was considered adequate to meet the requirement for protein, including essential amino acids.

In 2007, WHO/FAO/UNU published their most recent protein requirement (4), also based on the Rand meta-analysis (3), but with increased requirements for most essential amino acids, which made a certain level of protein quality necessary, and in 2012 the European Food Safety Authority (EFSA) published their Population Reference Intake for protein based on N-balance studies (5), again mainly the Rand meta-analysis (3). Both WHO and the EFSA Panel also considered several health outcomes associated with protein intake, but data were found to be insufficient to establish dietary reference values.

In 2007, the World Cancer Research Fund published a comprehensive report about the relationship between food, nutrition, and the prevention of cancer based on systematic literature reviews (6). The scientific recommendations were mainly based on foods/food groups (e.g. meat) and not on protein as a nutrient.

To date, recommendations on protein requirements have been based on N-balance studies, and recommendations of an optimal protein intake in relation to health outcomes are not clear. Except for the review regarding nutrition and cancer (6), the present evidence on the relationship between protein intake and health outcomes has, however, not been based on systematic literature reviews.

The purpose of this systematic review is to assess the evidence behind the dietary requirement of protein based on N-balance studies and to assess the health effects of varying protein intake in human nutrition based on prospective observational cohort studies, case-control studies, and randomized controlled studies.

## Methods

The process for conducting the systematic review is described in detail in the guidelines devised by the NNR5 working group (7). Briefly, the key characteristics of the systematic review are:

- Definition of the research questions to be answered.
- Definition of the eligibility criteria.
- A systematic search that attempts to identify all studies that would meet the eligibility criteria.
- A systematic selection and evaluation of the included papers.
- Construction of summary tables of the studies.
- Rating the evidence and formulate conclusions.

### Research questions

The research questions were formulated in cooperation with other relevant expert groups. The effects or associations marked with * should be reviewed in cooperation with or in the relevant expert groups (e.g. infants and children, elderly, pregnant and lactating women).

1. What is the *dietary requirement* of protein and protein of different dietary sources for adequate growth, development, and maintenance of body functions, mainly based on N-balance studies?
2. What is the association and what are the effects of different intake, timing, and frequency of protein and protein of different dietary sources, while considering the intake of other energy-giving nutrients at the same time, on:
  *well-established markers or indicators* of functional or clinical outcomes, such as serum lipids, glucose and insulin, blood pressure (BP), body composition, and bone mineral density (BMD)?
*functional or clinical outcomes* including
  - pregnancy* or birth outcomes*, growth, development, and sarcopenia*
  - cardiovascular diseases, weight outcomes, cancer, type 2 diabetes (T2D), fractures, renal outcomes, physical training, muscular strength, and mortality
3. Does intake and dietary source of protein (including vegan diet) affect the lactation/milk production in Nordic countries in relation to lactation duration, infant's need, and growth?*

### Eligibility criteria

We included studies with protein intake from foods, but excluded studies with isolated protein as supplements, and studies based on the intake of amino acids. The protein intake could be expressed as animal protein, vegetable protein and/or total protein (animal + vegetable).

#### Population

Studies of a generally healthy population in settings similar to the Nordic countries were included. Studies without Caucasians or with Caucasians as a minority group were excluded. Secondary prevention studies (e.g. hypertension stage 1 or hyperlipidemia with total cholesterol >6 mmol/L) were excluded, while studies including analyses on pre-hypertension (systolic BP of 120–139 mmHg or diastolic BP of 80–89 mmHg) were included, since this is a group of individuals at high risk of hypertension, justifying special attempts to lower BP. We also excluded studies of adiposity or obesity, and athletes.

#### Study type and design

Observational studies: prospective cohort studies and case-control studies were included, while cross-sectional studies were excluded. Studies were also excluded if length of follow-up was clearly too short related to outcome.

Controlled intervention studies: required length of study depended on the outcome; for N-balance studies the length was set to at least 14 days in accordance with a recent meta-analysis (3). Single-meal postprandial studies (acute studies) were excluded. The required number of participants depended on the outcome and power calculations.

Publication language was English or any of the Nordic languages.

#### Publication type

Original articles, meta-analyses, and systematic reviews were included. Narrative reviews were examined to ensure that all relevant studies were included.

#### Time period for publication

2000 up to and including 2011

### Search method and terms

The search terms were established in collaboration with a librarian and are shown in Appendix A. The databases used were PubMed and SweMed (the latter was used to identify Nordic papers not published in PubMed). The main search included the period January 2000–January 2011. An additional search was run in Medline through the PubMed platform in January 2012 in order to update the search with the most recent papers published from January to December 2011.

### Selection and evaluation of papers

The 5,718 abstracts from the initial search were screened by one of the authors (ANP) in order to exclude the clearly ineligible abstracts and to select abstracts that should be directed to the other expert groups. This left 1,483 abstracts to be screened in pairs by the three members of the protein expert group. In July 2011, a member of the initial expert group resigned. The two remaining experts (ANP and EB) made the *first screening*. All articles suggested by at least one of the two were ordered as full-text papers. In August 2011, the expert group was supplemented with a third expert (JK) who participated in the *second screening* of the full-text papers. The experts made the second screening in pairs, and papers suggested by at least one expert were included in the quality assessment. The *quality assessment* was done according to the principles in the guidelines (7). Briefly, a quality assessment tool specific for the study type was used to grade the papers as A (high-quality study with very low level of potential bias); B (some bias, but not enough to invalidate the results); C (significant bias and weaknesses that may invalidate the results). After the quality grading, *evidence tables* were constructed with a description and the quality assessment of each study. Finally, for each evaluated outcome the grading of evidence was based on *summary tables* and a four-class grading: convincing (high), probable (moderate), suggestive (low), and no conclusion (insufficient). The minimum requirement for ‘suggestive’ was two studies showing an association and no conflicting results. If some studies showed a non-significant (neither positive nor negative) association, it was decided that for ‘suggestive evidence’ the number of results showing an association was required to be at least two times higher than those showing no association.

## Results

**Fig. 1:**
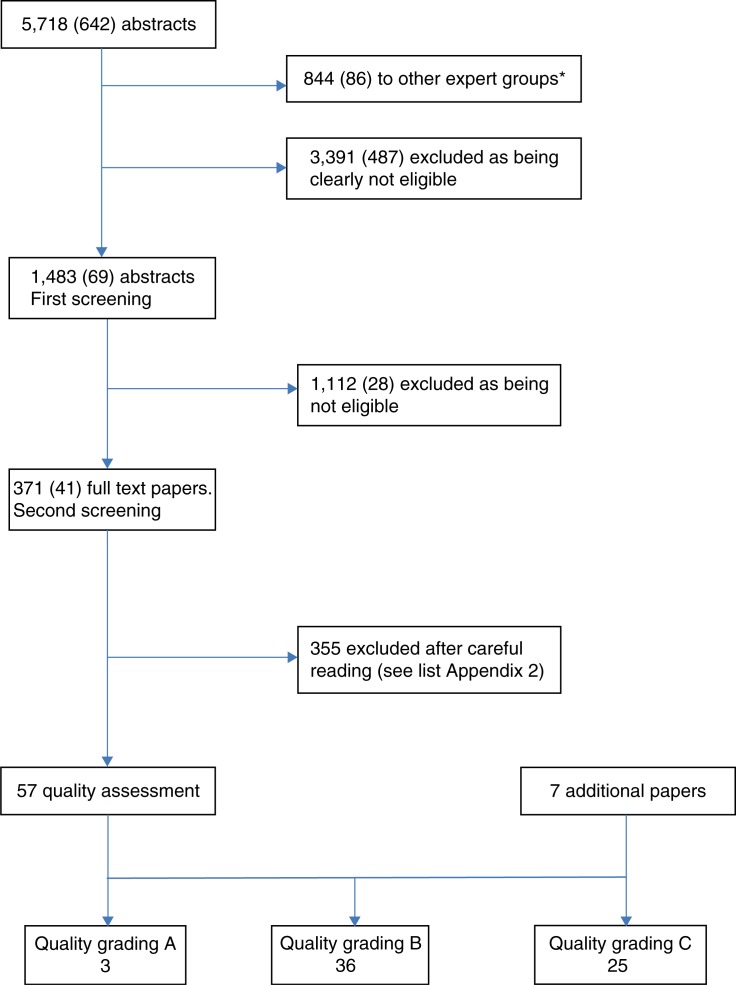
Flow chart of the systematic literature review process. Numbers in brackets are the additional search in 2011. *Some of the abstracts are both sent to other groups and kept in the protein group.

The included 1,483 abstracts were initially screened for eligibility (Fig. 1). Of these, 371 were selected and ordered as full-text papers, including narrative reviews. The search in SweMed resulted in 113 abstracts, and none of them were ordered as full-text papers.

The additional search resulted in 642 abstracts of which 487 were excluded as being clearly ineligible, 86 were directed to the other expert groups, and 69 were screened in pairs. Of these, 41 were ordered as full-text papers. Thus, a total of 412 full-text papers were ordered.

After careful scrutiny, 64 papers were quality graded, including 7 additional papers identified through reference lists from the included papers and the narrative reviews.

The reasons for exclusion of the 355 full-text papers are shown in Appendix B.

### Dietary requirement based on N-balance studies

The studies used for the grading of evidence for protein requirements based on N-balance studies are a meta-analysis including 19 balance studies (3), a controlled metabolic study of three 18-day periods (8), and a controlled single blinded short-term study with high versus usual protein (UP) intake (9), quality graded as B, A, and B, respectively (see Appendix C, Table C1).

Rand et al.'s meta-analysis from 2003 (3) included 19 N-balance studies of eucaloric diets with at least three test protein intakes. They found no significant differences in requirements between adult age, sex, or source of dietary protein, but they also stated that the data did not provide sufficient power to detect possible differences. The median-estimated protein requirement of good-quality protein was 0.66 g per kg body weight (BW) per day, and the estimated recommended dietary allowance (RDA) was set to 0.83 g good-quality protein/kg BW per day (97.5th percentile). Campbell et al. (8) tested young versus old, and men versus women in a controlled metabolic study with a low-protein (0.5 g/kg BW), medium-protein (0.75 g/kg BW), and high-protein (HP) (1 g/kg BW) diet. The N-balance was not different between the four groups, and the estimated requirement expressed per kg BW was not significantly different for the young versus old or men versus women. Mean protein requirement was lower for older women versus older men, but when expressed per kg fat-free mass (FFM), there was no significant difference. For all subjects combined, the adequate protein allowance was estimated to be 0.85±0.21 g/kg BW per day and not statistically different from the estimate of 0.83 g/kg BW per day, as suggested by Rand et al. (3).

A short-term study was also included because of an HP intake in the test meal versus UP intake (9). Young men and women (UP: 1.04 g/kg BW and HP: 2.08 g/kg BW) versus old men and women (UP: 0.89 g/kg BW and HP: 1.79 g/kg BW) were tested for 10 days on each diet in a cross-over design. There was no age-related difference in N-balance. Nevertheless, there was concern about an HP diet corresponding to ca. 24 E% in the elderly because of a potentially negative effect on the kidney function expressed as a lack of increase in glomerular filtration rate (GFR) from a reduced value among the elderly.

In summary, the evidence is assessed as *probable* regarding the estimated average requirement and the subsequent RDA of 0.83 g good-quality protein/kg BW per day (Table 1). The evidence of potential adverse effects of an HP diet (ca. 24 E%) is regarded as *inconclusive* (Table 1).

**Table 1:** Summary table N-balance studies

Exposure/Intervention	Outcome variable	Study	Number of participants (age) Men (M), Women (W)	Effect of protein	Rating A B C	Strength of evidence: Convincing, probable, Suggestive, no conclusion
	N-balance	Meta-analysis (3)	235 M and W in 19 separate studies	EAR: 0.66 g/kg body weight RDA: 0.83 g/kg BW	B	Probable
Low protein (0.5 g/kg) Medium (0.75 g/kg) High (1.0 g/kg)	N-balance	Controlled metabolic study (8)	23 young and 19 old M and W	Estimated RDA: 0.85 g/kg BW	A	

Usual protein: 1.5 g/kg FFM (1 1–12 E%) High protein: 3 g/kg FFM (22–24 E%)	N-balance, glomerular filtration rate (GFR)	Controlled cross-over study (9)	10 young and 9 old M and W	N-balance not different between young and old and between men and women. GFR was lower in older participants and they had a lesser adaption response to the HP diet	B	No conclusion

### Protein intake and mortality

The evaluation of the association between protein intake and mortality among healthy individuals is based on seven prospective cohort studies with nine populations included (10–16), four papers quality graded as B (10, 14–16), and three papers quality graded as C (11–13) (see Appendix C, Table C2). Such an association might be expected as a result of a possible association between protein intake and cancer or cardiovascular diseases, as described in the following sections.

Fung et al. (10) used pooled data from the Nurses’ Health Study (NHS) and Health Professionals’ Follow-up Study (HPFS) and, based on a food frequency questionnaire (FFQ), they created a low-carbohydrate (LC) score from deciles of the energy percentage (E%) of fat, protein, and carbohydrate (CH). They also made an animal LC score (animal fat and animal protein) and a vegetable LC score (vegetable fat and vegetable protein). The range of intake of total protein was ca. 15–23 E%. They found that a high overall low-carbohydrate–high protein (LCHP) score was associated with an increase in all-cause mortality, and that a high LCHP score based on animal protein and animal fat, was even more positively associated with all-cause mortality, cardiovascular disease mortality, and cancer mortality, while the LCHP score based on vegetable protein and vegetable fat was associated with lower all-cause mortality and cardiovascular disease mortality. Thus, the health effects of LC diets may depend on the sources of protein and fat. The authors also emphasized that the presented LC scores were not designed to mimic any particular versions of the LC diets in the popular literature, and therefore the risk estimates did not correspond with any versions of LC diets in the population.

In the Prevention of Renal and Vascular ENd-stage Disease (PREVEND) study (11), the focus was on mortality, cardiovascular events, and renal outcomes. The protein intake was calculated from two 24-h urinary urea excretions and expressed as protein intake in g/kg ‘ideal’ BW, i.e. after correcting BW to a body mass index (BMI) corresponding to 22. Thus, the level of protein intake could not be assessed, because the correction probably overestimated intakes, and because of no correction for possible loss of urine in the collections. They found quintiles of protein intake inversely associated with all-cause mortality and non-cardiovascular mortality.

In the Iowa Women's Health Study (12), total, animal and vegetable protein E% in quintiles from an FFQ were isoenergetically substituted for CH. The range of intake was from 14 E% in the lowest quintile to 22 E% in the highest. No association between the intake of total and animal protein, respectively, and mortality (all-cause, coronary disease mortality and cancer mortality) was observed in the multivariate models. Vegetable protein was inversely associated with coronary disease mortality, and substituting vegetable protein for animal protein was also inversely associated with coronary disease mortality.

The Swedish Women's Lifestyle and Health cohort study (13) used energy-adjusted increasing protein and decreasing CH intake in deciles, and a combination of them in an LCHP score. The range of protein intake in deciles was from 10 to 23 E%. Increasing protein intake was associated with increased risk of cardiovascular disease mortality, and the combination score was even more predictive. The combination score was also positively related to increased risk of all-cause mortality. The associations were more pronounced for cardiovascular mortality in women aged 40–49 years at baseline compared to women aged 30–39 years at baseline. The dietary assessment was based on an FFQ and the mean energy intake of ca. 6.4 MJ indicated under-reporting. The authors emphasized that the presented LCHP score did not address the potential short-term effects of LCHP diets in the control of BW or insulin resistance, but they did draw attention to the potential long-term adverse health effects of a diet generally low in CHs and high in protein, especially with respect to cardiovascular health.

Based on FFQ, The Health Professionals’ Follow-Up Study examined quintiles of protein E% (total, animal, and vegetable) substituted for an isocaloric amount of CHs and the association with fatal and non-fatal ischemic heart disease (IHD). An inverse relationship of vegetable protein to fatal IHD was found (14).

Prentice et al. (15) pooled two cohorts from the Women's Health Initiative, and from FFQ they used ‘biomarker calibrated’ protein intake in gram per day and protein E%. They found protein E% inversely related to coronary heart disease mortality, while the relation to protein intake in gram per day was non-significant.

In the Greek cohort of the European Prospective Investigation into Cancer and Nutrition (EPIC) study, Trichopoulou et al. (16) evaluated the association between mortality and a habitual LCHP diet expressed as a score based on deciles of energy-adjusted intake. The range of intake of total protein was ca. 10–20 E%. The LCHP score was positively related to all-cause mortality, and expressed as a 2-unit increase, also positively related to cardiovascular deaths. The dietary assessment was based on an FFQ and under-reporting was particularly present among women. The authors emphasized that the data evaluated the health consequences of long-term habitual dietary intakes and should not be interpreted as indicating that short-term use of LCHP diets is detrimental to health.

For mortality, the relationship between protein intake *per se* and all-cause mortality is regarded as *inconclusive*, while the evidence is assessed as *suggestive* regarding an increased risk of all-cause mortality in relation to an LCHP diet with total protein intake of at least 20–23 E% in three studies, including four prospective cohorts (Table 2).

For cardiovascular mortality, the evidence is assessed as *suggestive* for an inverse relation to vegetable protein intake based on three studies with four prospective cohorts (Table 2).

**Table 2:** Summary table mortality

				Association of protein/effect (in RCT)					
			Number of participants (age) Men (M), Women (W)					Rating A B C	Strength of evidence: Convincing, probable, Suggestive, no conclusion
Exposure/Intervention	Outcome variable	Study		Total	Animal	Vegetable	A/V ratio		
Low-carbohydrate (LC) score, animal-based or vegetable-based	All-cause mortality	Pooled analysis of two cohorts (10): Nurses’ Health Study	85,168 (34–59 years) W	POS	POS	INVERSE	NA	B	No conclusion for total protein intake
		Health Professionals Study	44,548 (40–75 years) M						Suggestive for an LCHP diet
Continuous values and quintiles of estimated baseline protein intake in g/kg body ‘ideal’ weight (BW) (after correcting the BMI to 22)	All-cause mortality	Cohort (11)	5,778 (mean 50 years) M and W	INVERSE	NA	NA	NA	C	
Protein E% in quintiles	All-cause mortality	Cohort (12)	29,017 (55–69 years) W	NS	NS	NS	NS*	C	
Energy-adjusted: 1) increasing protein in deciles (and decreasing CH intake), 2) and a combination (LCHP score)	All-cause mortality	Cohort (13)	42,237 (30–49 years) W	NS POS	NA NA	NA NA	NA NA	B	
A low carbohydrate – high protein score (LCHP) using deciles of energy-adjusted intake	All-cause mortality	Cohort (16)	28,572 (20–86 years) M and W	POS	NA	NA	NA	B	

Low-carbohydrate (LC) score, animal based or vegetable based	Cardiovascular mortality	Pooled analysis of two cohorts (10): Nurses’ Health Study Health professionals Study	85,168 (34–59 years) W 44,548 (40–75 years) M	NS	POS	INVERSE	NA	B	Suggestive for vegetable protein including an LCHP diet based on vegetable protein
Protein E% in quintiles	Cardiovascular mortality	Iowa Women's Health Study (12)	29,017 (55–69 years) W	NS	NS	INVERSE	INVERSE*	C	
Energy-adjusted: 1) increasing protein in deciles (and decreasing carbohydrate intake), 2) and a combination (LCHP score)	Cardiovascular mortality	The Women's Lifestyle and Health Cohort (13)	42,237 (30–49 years) W	POS POS	NA NA	NA NA	NA NA	C	
A low carbohydrate – high protein score (LCHP) using deciles of energy adjusted intake	Cardiovascular mortality	The Greek cohort of EPIC (16)	22,944 (20–86 years) M and W	POS	NA	NA	NA	B	
Quintiles of energy percentage (E%) protein (total, animal and vegetable), substitution of protein for an isocaloric amount of carbohydrate (CH)	Fatal IHD	Health Professionals Follow-Up Study (14)	43,960 (40–75 years) M	NS	NS	INVERSE	NA	B	
Biomarker “Calibrated”:	Cardiovascular	Two cohorts from the	80,370 W					B	
1) Protein in gram per day 2) and protein E%	mortality	Women's Health Initiative (15)		NS INVERSE	NA NA	NA NA	NA NA		

Low-carbohydrate (LC) score, based or vegetable based animal	Cancer mortality	Pooled analysis of two cohorts (10): Nurses’ Health Study	85,168 (34–59 years) W	NS	POS	NS	NA	B	No conclusion
		Health Professionals Study	44,548 (40–75 years) M						
Protein E% in quintiles	Cancer mortality	Iowa Women's Health Study (12)	29,017 (55–69 years) W	NS	NS	NS	NS*	C	
Energy-adjusted: 1) increasing protein in deciles (and decreasing CH intake), 2) and a combination (LCHP s core)	Cancer mortality	The Women's Lifestyle and Health Cohort (13)	42,237 (30–49 years) W	NS NS	NA NA	NA NA	NA NA	C	
LCHP score using deciles of energy adjusted intake	Cancer mortality	The Greek cohort of EPIC (16)	22,944 (20–86 years) M and W	NS	NA	NA	NA	B	

* vegetable protein substituted isoenergetically for amount of animal protein.

Regarding protein intake and the sources of protein (animal versus vegetable) and the relation to cancer deaths, the evidence is assessed as *inconclusive* (Table 2).

### Protein intake and cancer

The evaluation of the association between protein intake and breast cancer is based on one prospective cohort study (17) and two nested case-control studies (18, 19), quality graded as C, B, and C, respectively (see Appendix C, Table C3). A possible association with cancer could be explained by an increased production of growth factors, such as insulin-like growth factor I (19) and/or the formation and absorption of carcinogens produced during cooking or processing of meat (17).

In the Nurses’ Health Study (17), there were no statistically significant associations between breast cancer and total, animal or vegetable protein intake, based on FFQ. In a nested case-control study by Sala et al. (18), the odds ratio of having a high-risk mammographic parenchymal pattern in the highest tertile of total protein intake, based on 7-day diaries, was twice that of women in the lowest tertile, while an Italian nested case-control study (19) found no relationship between breast cancer and total, animal or vegetable protein, based on FFQ, but with no information about the total energy intake.

Thus, the evidence is assessed as *inconclusive* regarding the relation of protein intake to risk of breast cancer (Table 3).

**Table 3:** Summary table cancer

				Association of protein/effect (in RCT)				
			No. of participants (age) Men (M), Women (W)				Rating A B C	
Exposure/Intervention	Outcome variable	Study		Total	Animal	Vegetable		Strength of evidence: Convincing, probable, Suggestive, no conclusion
Quartiles of energy percentage (E%) of total, animal, and vegetable protein	Breast cancer	Nurses’ Health Study (17)	88,647 (mean 46.7 years) W	NS	NS	NS	C	No conclusion
Total protein in g per day in tertiles	Mammographic parenchymal patterns	EPIC-Norfolk and the National Health Service	203 cases 203 controls	POS	NA	NA	B	
		Regional Breast Screening						
		Programme for Norwich (18)						
Energy adjusted intake in tertiles of total, animal and vegetable protein	Breast cancer	ORDET Cohort (19)	56 cases 214 controls	NS	NS	NS	C	

Animal protein in gram per day	Colorectal cancer	Meta-analysis of 3 cohort studies and 3 case-control studies (20)	1,070 cases and app. 1.5 million person years	NA	NS	NA	C	No conclusion
Total and animal protein in gram per day	Colorectal adenomas (high malign potential)	Case-control study (21)	87 cases 35 hospital controls 35 healthy controls	NS	NS	NA	B	
Quintiles of total protein intake in gram per day	Colorectal adenomas (high malign potential)	Case-control study (22)	182 cases 178 hospital controls 182 healthy controls	NS	NA	NA	C	
Energy adjusted total protein intake in tertiles	Colon cancer	Case-control study (23)	286 cases 550 controls	NS	NA	NA	C	

Energy adjusted intake in quintiles of total, animal and vegetable protein in gram per day	Laryngeal cancer	Case-control study (24)	527 cases 1,297 controls	POS	POS	INVERSE	C	No conclusion
Energy adjusted intake in quartiles of total, animal and vegetable protein in gram per day	Non-Hodgkin's lymphoma	Case-control study (25)	601 cases 717 controls	NS	POS	NS	C	No conclusion
Energy adjusted intake in quartiles of total, animal and vegetable protein in gram per day	Esophageal and gastric cancer	Case-control study (26)	537 target cases 558 comparison case groups 687 controls	POS	POS	INVERSE	B	No conclusion
Energy adjusted intake in quartiles of total protein in gram per week	Ovarian cancer	Case-control study (27)	442 cases 2,135 controls	NS	NA	NA	B	No conclusion
Energy adjusted intake in quintiles of total, animal and vegetable protein in gram per day	Pancreatic cancer	Case-control study (28)	326 cases 652 controls	NS	POS	NS	C	No conclusion
Total protein gram per week and in quartiles	Prostate cancer	Case-control study (29)	1,797 cases 2,547 controls	NS	NA	NA	B	No conclusion
Total, animal and vegetable protein E% in quintiles	Renal cell cancer	Pooled analysis of 13 prospective cohort studies (30)	530,469 W 244,483 M	NS	NS	NS	B	No conclusion

The evaluation of the association between protein intake and colorectal cancer is based on one meta-analysis (20) and three case-control studies (21–23), quality graded as C, B, B, and C, respectively (see Appendix C, Table C4). A possible association between the protein intake and colorectal cancer is most commonly explained by a production of carcinogens arising either from cooking or from preservation of meat products (22).

In the meta-analysis (20), the main focus was on fat, and not protein, in relation to cancer. Some of the included studies regarding protein intake were not relevant in a Nordic setting, and only a few studies included animal protein, while most studies included foods (meat). They found no significant association between animal protein and colorectal cancer. Two of the case-control studies used colorectal adenomas (as precursors for colorectal cancer) as the outcome and used hospital controls as well as healthy controls. None of them found a statistically significant relation to total protein intake (21, 22) or animal protein intake (21). The third case-control study found no statistically significant association between colorectal cancer and total protein intake (23).

The evidence is assessed as *inconclusive* regarding the relation of protein intake to risk of colorectal cancer (Table 3).

The following associations between protein and cancer diseases are based on just one study (see Appendix C, Table C5) and thus regarded as *inconclusive* (Table 3). All of the studies used FFQ.

The evaluation of the association between protein intake and laryngeal cancer is based on an Italian case-control study with hospital controls, quality graded as C (24). They found a statistically significant increased risk for total and animal protein intake and a slightly reduced risk with increased vegetable protein intake.

The evaluation of the association between protein intake and non-Hodgkin's lymphoma is based on a US case-control study with population-based controls, quality graded as C (25). They found a statistically significant increased risk with increasing animal protein intake and a reduced risk with increased vegetable protein intake.

The evaluation of the association between protein intake and esophageal and gastric cancer is based on a US case-control study with population-based controls, quality graded as B (26). They found a statistically significant increased risk with increased intake of total and animal protein and a reduced risk with increased vegetable protein intake.

The evaluation of the association between protein intake and ovarian cancer is based on a Canadian case-control study with population-based controls, quality graded as B (27). They found no statistically significant association with total protein intake.

The evaluation of the association between protein intake and pancreatic cancer is based on an Italian case-control study with hospital controls, quality graded as C (28). They found a statistically significant increased risk related to animal protein intake, but no statistically significant associations with total and vegetable protein intake.

The evaluation of the association between protein intake and prostate cancer is based on a Canadian case-control study with population-based controls, quality graded as B (29). They found no statistically significant association with total protein intake.

The evaluation of the association between protein intake and renal cell cancer risk is based on a pooled analysis of 13 prospective cohort studies (30), quality assessed as B. They found no statistically significant associations with the E% of total, animal or vegetable protein in quintiles. The intake level of the quintiles was not reported, thus the intake cannot be assessed.

### Protein intake and cardiovascular disease

The evaluation of the association between protein intake and coronary heart disease is based on four prospective cohort studies (11, 14, 15, 31), quality graded as C, B, B, and B, respectively (see Appendix 3, Table C6). It has been suggested that the possible association between cardiovascular disease and protein intake is caused by the effect of protein intake on BP, plasma LDL, and weight maintenance (14).

In the PREVEND study (11), the focus was on mortality, cardiovascular events, and renal outcomes. As mentioned earlier, the protein intake was calculated from two 24-h urinary urea excretions and expressed as protein intake in g/kg ‘ideal’ BW, i.e. after correcting BW to a BMI corresponding to 22. Thus, the level of protein intake could not be assessed, because the correction probably overestimated intakes, and because of no correction for possible loss of urine in the collections. They reported a statistically significant (non-linear) relationship between protein intake, as a continuous variable, and cardiovascular events, based on a Cox regression analysis corrected for confounding variables. However, we could not reproduce the reported statistical significance of the association between protein intake in quintiles and cardiovascular events according to their Table 2, and we have therefore chosen not to include this association in our table and conclusion.

Based on FFQ, The Health Professionals Follow-Up Study (14) examined quintiles of protein E% (total, animal, and vegetable) substituted for an isocaloric amount of CHs and the association with fatal and non-fatal IHD. The lowest quintile of total protein was 14.6 E% and the highest quintile 22.5 E% substituted for CH. They found no association between protein E% and risk of total IHD (non-fatal and fatal), but a higher intake of total and animal protein E% was associated with increased risk of IHD in a subgroup of ‘healthy’ men (without baseline hypertension, diabetes, and hypercholesterolemia).

Based on FFQ, the Nurses’ Health Study (31) used a low-CH score (low CH and high fat and HP diet) from energy percentages, and they also separated protein and fat into animal- and vegetable-based sources. They found an inverse relation to coronary heart disease when the score was based on vegetable sources. In separate analyses of each macronutrient, no statistically significant associations were found between total, animal, or vegetable protein and coronary heart disease.

Two cohorts from the Women's Health Initiative study were pooled in the analysis by Prentice et al. (15), where they used ‘Biomarker Calibrated’ protein intake and protein E%. They found that protein E% was associated with an increased risk of coronary heart disease.

The evidence is assessed as *inconclusive* regarding the relationship between protein intake and risk of coronary heart disease (Table 4).

**Table 4:** Summary cardiovascular disease

				Association of protein/effect (in RCT)				
			Number of participants (age) Men (M), Women (W)				Rating A B C	
Exposure/Intervention	Outcome variable	Study		Total	Animal	Vegetable		Strength of evidence: Convincing, probable, Suggestive, no conclusion
Low-carbohydrate (CH) score (low CH and high fat and high protein diet) based on energy percentages (E%)	Fatal and non-fatal coronary heart disease	Cohort (31)	82,802 W	NS	NS*	INVERSE**	B	No conclusion
Also protein E% (total, animal and vegetable) in a separate analysis				NS	NS	NS		
Quintiles of E% protein (total, animal and vegetable), substituted for an isocaloric amount of carbohydrate (CH)	Ischemic heart disease	Cohort (14) In a subgroup of “healthy” men	43,960 M	NS POS	NS POS	NS NS	B	
“Calibrated” Protein intake and protein E%	Coronary heart disease (AMI)	Cohort (15)	80,370 W	INVERSE	NA	NA	B	

Quintiles of energy adjusted total, animal and vegetable protein in gram per day	Total strokes Intraparenchymal hemorrhages	Cohort (32)	85,764 W	NS NS	NS INVERSE	NS NS	C B	No conclusion
Quintiles of E% protein (total, animal and vegetable), substituted for an isocaloric amount of carbohydrate (CH)	Fatal and non-fatal strokes	Cohort (33)	43,960 M	NS	NS	NS	B	

A diet with 15 E% protein vs. 25 E% protein, and the 10 E% protein replaced with carbohydrate	Blood pressure	Randomized cross-over feeding study (34)	164 M and W	NS***	NA	NA	B	No conclusion for total and animal protein Suggestive for vegetable protein
Quintiles of energy percentage (E%) of total, animal and vegetable protein	Hypertension	Cohort (35)	5,880 M and W	NS	NS	INVERSE	B	
E% of total, animal and vegetable protein	Blood pressure	Cohort (36)	1,714 M	NS	NS	INVERSE	B	
Energy percentage (E%) or gram per day of total, protein intake	Systolic and diastolic blood pressure	Meta-analysis (37)	19,954 M 950 W 12,508 M and W	NEG	NA	NA	C	
Intake of soya protein in gram per day	Systolic and diastolic blood pressure	Meta-analysis (38)	1,608 M and W	NA	NA	INVERSE	B	
Daily intake of app. 25 g soya protein (range: 15–40 g)	Total cholesterol	Meta-analysis (39)	2,913 M and W	NA	NA	INVERSE	B	Probable to convincing for soya protein on LDL-cholesterol
	LDL-cholesterol			NA	NA	INVERSE		
	HDL-cholesterol			NA	NA	NS		
	TG			NA	NA	INVERSE		
Soya consumption vs. nonsoya control diets, less than 65 g soy protein/day median 30 g/day	LDL-cholesterol HDL-cholesterol	Meta-analysis (40)	43 RCTs with 59 treatment arms	NA NA	NA NA	INVERSE INVERSE	A	

* score based on animal sources of protein and fat.

** score based on vegetable sources of protein and fat.

*** in a subgroup analysis with only Caucasians.

The evaluation of the association between protein intake and fatal/non-fatal strokes is based on two prospective cohort studies (32, 33), quality graded as C and B, respectively (see Appendix C, Table C7).

In the Nurses’ Health Study (32), they used quintiles of energy-adjusted total, animal, and vegetable protein in gram per day based on FFQ, and found no relation to strokes. However, in the subgroup with intraparenchymal hemorrhages, the risk was inversely associated with animal protein.

Based on FFQ, the Health Professionals’ Follow-Up Study (33) examined quintiles of protein E% (total, animal, and vegetable) substituted for an isocaloric amount of CHs and the association with fatal and non-fatal strokes. The lowest quintile of total protein was 14.6 E% and the highest quintile 22.5 E% substituted for CHs. They found no association between protein E% from any source of protein substituted for CH and risk of total strokes (non-fatal and fatal).

The evidence is assessed as *inconclusive* regarding the relation of protein intake to risk of stroke (Table 4).

The association between protein intake and BP is based on one feeding study (34), quality graded as B, two prospective cohort studies (35, 36), both quality graded as B, and two meta-analyses (37, 38), quality graded as C and B, respectively (see Appendix C, Table C8).

The OmniHeart randomized trial compared the effect of healthy diets with partial substitution of CHs with either protein (about half from vegetable sources) or monounsaturated fat in adults with pre-hypertension or hypertension stage 1 (34). In a subgroup analysis of the 40% Caucasians, there was a statistically non-significant inverse association between the protein diet partially substituted for CHs. In a subgroup analysis of pre-hypertensive participants, the protein diet lowered BP significantly, but the analysis was not controlled for race (only 40% Caucasians) and BW (only 21% were not overweight or obese), and thus not comparable to a healthy Nordic population.

The Spanish SUN cohort of university graduates (35) found an inverse relationship between risk of hypertension and vegetable protein intake expressed in quintiles of energy-adjusted gram per day based on a Spanish version of FFQ. The Chicago Western Electric Study (36) used the dietary history method, and also found an inverse relationship between BP change and vegetable protein intake expressed in E%, but they did not control for potassium and fiber, and thus it is difficult to separate the influence of vegetable protein *per se*.

Liu et al.'s meta-analysis from 2002 (37) included nine cross-sectional studies and two prospective cohort studies (one study with adults and one with children). They found an inverse association between dietary protein intake and BP in men and women, and the association was dependent on the dietary assessment method. Evidence from the longitudinal studies was limited. A recent meta-analysis from 2011 (38) of 25 randomized controlled trials (RCTs) analyzed the effect of soya protein versus control on BP. Soya protein intake ranged between 18 and 66 g/day with a median of 30 g/day. The analysis found that soya protein reduced BP, and that the difference to the control groups was more pronounced in hypertensive groups, in trials using CH as the control diet versus casein/milk in the control diet, in parallel design, and with intervention duration of at least 12 weeks.

The evidence is assessed as *inconclusive* regarding the relation of total and animal protein intake to BP, but it is assessed as *suggestive* regarding the inverse relation of vegetable protein to BP (Table 4).

The evaluation of the effect of protein on serum lipoprotein risk factors for coronary heart disease is based on two meta-analyses of RCTs with soya protein (39, 40), quality graded as B and A, respectively (see Appendix C, Table C9).

The meta-analysis from 2008 (39) of 30 RCTs covered the period 1995–2007. They concluded that ‘the inclusion of modest amounts of soya protein (ca. 25 g) into the diet of adults with normal or mild hypercholesterolemia resulted in a small, highly significant reduction in total and LDL cholesterol equivalent to ca. 6% LDL reduction’. The most recent meta-analysis of 43 RCTs also included the impact of study design (40). They found that parallel studies scored higher in study quality than cross-over studies, and that the parallel RCTs were associated with significantly greater improvements in LDL values. A sub-analysis also showed that studies with highest baseline LDL had greater reductions than studies with the lowest values. Thus, the effect may be smaller in normocholesterolemic subjects. Overall, they found that 15–30 g soya protein (1–2 servings per day) had a positive impact on LDL cholesterol.

The evidence is assessed as *probable* to *convincing* regarding the effect of soya protein on LDL cholesterol (Table 4).

### Bone health

The evaluation of the association between protein and bone health is based on five prospective cohort studies, two randomized controlled studies, and two systematic reviews and meta-analysis (see Appendix C, Table C10). Such an association could be explained by the effect of protein-associated acid load or effect of protein intake on calcium retention and/or increases in insulin-like growth factor I (47, 49).

Three cohort studies (41–43), all quality graded as C, and a systematic review and meta-analysis (44), quality graded as B, were identified based on the association between protein and BMD or bone loss. The cohort studies included young women, postmenopausal women, and older men and women. In the cohort study of young women (41), the main focus was on contraceptive use in relation to BMD, while the relation to protein intake was a secondary analysis. Based on FFQ, they found no longitudinal effect of total, animal, and vegetable protein E% on changes in BMD. In the cohort of postmenopausal women (42), they found higher bone loss related to a higher animal/vegetable (A/V) protein ratio and also no relation to total protein intake after 7 years follow-up. The women had a median protein intake of 17 E%, but the intake data were weakened by a very low reported energy intake (mean ca. 5 MJ), estimated from FFQ. Among older men and women in the Framingham Osteoporosis Study (43), a lower E% of total and animal protein was associated with a higher bone loss after 4 years, and the highest quartile of total protein intake (1.2–2.8 g/kg BW) was associated with lower bone loss. They used an FFQ, and there was no information about the total energy intake. The systematic review and meta-analysis (44) concluded that the overall impression was a small benefit of protein on bone health based on cross-sectional and supplemental studies. The analysis was weakened by limited information about the quality of the dietary assessment methods.

The evidence is assessed as *inconclusive* regarding the relation of protein intake to bone loss (Table 5).

**Table 5:** Summary bone health

				Association of protein/effect (in RCT)					
			Number of participants (age) Men (M), Women (W)					Rating A B C	
Exposure/Intervention	Outcome variable	Study		Total	Animal	Vegetable	A/V ratio		Strength of evidence (strong, medium, low) Suggestive No conclusion
Protein E% in tertiles	Bone loss	Cohort (41)	560 (14–40 years) W	NS	NS	NS	NA	C	No conclusion
Protein E% in tertiles and A/V ratio	Bone loss	Cohort (42)	1,035 (>65 years) W	NS	NA	NA	POS	C	
Total protein intake (g/kg BW) and protein E% in quartiles	Bone loss	Cohort (43)	615 (69–97 years) M and W	INVERSE INVERSE	INVERSE INVERSE	NA NA	NA NA	C	
Total protein intake in g/day or g/kg BW		Review and meta-analysis (44):						B	
	Bone loss	Cohort		NS					
	BMD	RC trials		POS					

Protein (g/kg BW)	Fracture	Cohort (45)	36,217 (40–65 years) W	NS	NA	NA	NA	B	No conclusion
Energy adjusted per 1,000 kcal of total, animal, and vegetable protein in tertiles:									
Low calcium				POS	POS	INVERSE	NA		
High calcium				NS	NS	NS	NA		
Protein (g/day) in tertiles for total, animal and vegetable	Fracture	Cohort (46)	3,656 (mean 55 years) M and W	NS	NA	NA	NA	B	
Low calcium				NS	POS	NS	NS		
High calcium				NS	INVERSE	NS	NS		
Protein E% in tertiles and A/V ratio	Fracture	Cohort (42)	1,035 (> 65 years) W	NS	POS	NS	POS	C	
Total protein intake in g/day or g/kg BW	Fracture	Review and meta-analysis (44): RC trials		NS				B	

Dietary acid load (including protein)	Osteoporosis	Review and meta-analysis (47)		NS				C	No conclusion

High and usual protein intake combined with high and low sodium diet High and low protein intake combined with high and low calcium diet	Calcium- and bone metabolism Calcium- and bone metabolism	Randomized cross-over trial (48) Randomized cross-over trial (49)	24 (50–67 years) W 27 (50–69 years) W	High protein-high sodium: increased calcium loss lead to increased bone resorption. Cannot separate protein and sodium effects POS interaction (high protein increased calcium retention when calcium intake was low)				B A	No conclusion

Three prospective cohort studies (42, 45, 46), quality graded as B, B, and C, respectively, and a systematic review and meta-analysis (44) quality graded as B, were identified based on the association between protein and risk of fractures (see Appendix C, Table C10).

Based on a validated FFQ, a French study of postmenopausal women with a habitual HP intake (45), there was no overall association between fracture risk and total protein intake. In the presence of low calcium intake (<400 mg/1,000 kcal), there was an increased risk of fractures related to energy-adjusted total and animal protein as well as gram per kg BW, while energy-adjusted vegetable protein was associated with a decreased fracture risk. In the Framingham Offspring Study of men and women (46), there was no overall association between fracture risk and total protein intake based on FFQ. Animal protein intake was associated with an increased fracture risk provided a low (<800 mg) calcium intake and a decreased risk of fractures provided a high (>800 mg) calcium intake. The Study of Osteoporotic Fractures in postmenopausal women (42) found increased risk of hip fractures related to high animal protein intake and high A/V ratio estimated from FFQ. When the model was adjusted for BMD, the relation of A/V ratio to fracture risk became non-significant. The systematic review and meta-analysis (44) found no relationship between protein intake and risk of fractures, neither in the cohort studies nor in the supplemental studies.

The evidence is assessed as *inconclusive* regarding the relation of protein intake to risk of fractures (Table 5).

A systematic review and meta-analysis assessed the relation of dietary acid load to bone health (47), quality graded as C because of the lack of information about dietary intake methods or intervention (see Appendix C, Table C10). The analysis did not support the hypothesis that ‘acid’ from the diet causes osteoporosis or that an ‘alkaline’ diet prevents osteoporosis. The systematic review also indicated that higher protein intake and animal protein were not detrimental to calcium retention. The ideal protein intake for bone health could not be determined.

The evidence is assessed as *inconclusive* regarding the relation of protein intake (acid load) to the risk of osteoporosis or calcium retention (Table 5).

Two intervention trials including postmenopausal women (48, 49), quality graded as B and A, respectively, were identified for the association between protein and calcium and bone metabolism (see Appendix C, Table C10). Harrington et al. (48) used a high-sodium–high-protein diet versus a low-sodium–UP diet in a randomized cross-over trial. Thus, it was difficult to separate the effect of protein *per se*. Nevertheless, they found that a high-sodium HP diet led to increased urinary calcium loss and increased bone resorption. In a high-quality feeding trial by Hunt et al. (49), high- (20 E%) or low- (10 E%) protein intake was combined with high- (1,510 mg) and low-calcium (675 mg) intake in a randomized four interventions’ cross-over design. They found that the combination of HP and low-calcium diet increased calcium retention, and it also resulted in an increase in IGF-1, an anabolic peptide hormone stimulating bone formation.

The evidence is assessed as *inconclusive* regarding the relation of protein intake to an overall effect on calcium and bone metabolism at normal intakes of sodium and calcium.

### Energy intake

The evaluation of the association between protein and energy intake is based on one prospective cohort study (50) and two intervention studies (51, 52), all quality graded as B (see Appendix C, Table C11). Such an association could be explained by a satiating effect of protein (51).

In the Amsterdam Growth and Health Longitudinal Study (50), 350 men and women were studied at the ages of 13, 32, and 36 years by the use of a dietary history method. During the 23-year follow-up, there was a decrease in energy intake of 125 kJ for men and 152 kJ for women for every increase in protein E% intake. The association between protein and energy intake was about three times stronger than the association between fat and energy intake. In this paper, the energy intake was only reported at the age of 36. Rumpler et al. (51) measured energy intake in 12 men during *ad libitum* food intake of two out of three treatments in two 8-week periods: drinks, based on foods providing 2.1 MJ, were included in a high-CH, high-fat, or HP diet. The HP diet included an additional 27 g protein/day. After the 8-week periods, there was no change in the energy intake for any of the macronutrients. In the discussion, the authors mentioned the possibility that the addition of 27 gram protein might have been insufficient to induce a change in the energy intake. In a strictly controlled intervention study of 19 weight-stable men and women (52), a weight-maintaining diet with 15 E% protein for 2 weeks was compared to a weight-stable diet with 30 E% protein in 2 weeks followed by an *ad libitum* diet for 12 weeks with 30 E% protein. The CH content of the diets was kept constant and thus, the fat content varied considerably, from 20 to 35 E%. The energy intake was unchanged during the weight-maintaining and weight-stable periods, but decreased during the 12-week *ad libitum* HP diet resulting in a significant weight loss of 4.9±0.5 kg. Since they tested an HP-low-fat diet, it was difficult to separate the effect of protein *per se*.

The evidence is assessed as *inconclusive* regarding the relation of protein to change in energy intake (Table 6).

**Table 6:** Summary energy intake

				Association of protein/effect (in RCT)				
			Number of participants (age) Men (M), women (W)				Rating A B C	
Exposure/Intervention	Outcome variable	Study		Total	Animal	Vegetable		Strength of evidence: Convincing, probable, Suggestive, no conclusion
Total protein intake (E%)	Energy intake	Cohort (50)	168 M 182 W	INVERSE	NA	NA	B	No conclusion
Addition of app. 27 g protein per day	Energy intake	RCT (51)	12 M	NS	NA	NA	B	
High protein-low fat diet:	Energy intake	Controlled trial (52)	19 M and W	INVERSE	NA	NA	B	
15 E% protein vs. 30 E%								

### BW control and body composition

Overall, the evaluation of the association between protein and BW and body composition is based on seven prospective cohort studies (50, 53–58), quality graded as C, C, B, B, B, B, and C, and two intervention studies (52, 59), quality graded as C and B, respectively (see Appendix C, Table C12). Such an association could be explained by a protein-induced increased thermogenesis and satiety (56).

Regarding the evidence of a relationship between protein intake and changes in BMI, two of the cohort studies and one controlled trial were used. In college students (53), a very simple frequency question about protein consumption per day was not associated with a 1-year change in BMI. Based on the dietary history method, the Chicago Western Electric Study (54) found that quartiles of animal E% protein intake was positively related to risk of overweight (BMI ≥ 25) and obesity (BMI ≥ 30), while vegetable protein was inversely related to obesity among men aged 40–55 years at baseline. In an RCT, 15 physically active men were prescribed an HP diet: 1.9 g/kg BW (22 E%) versus a normal diet (NP): <1.3 g/kg BW (15 E%) in 6 months (59). The main focus and power calculation were on vascular reactivity, but they also measured BMI and body composition and found no statistically significant effect on association with BMI despite a significant decrease in BW of 2 kg in HP (3.5% of baseline BW) versus 0.7 kg in the NP group (1% of baseline BW).

The evidence is assessed as *inconclusive* regarding the relation of protein intake to change in BMI (Table 7).

**Table 7:** Summary body weight and body composition

				Association of protein/effect (in RCT)				
			Number of participants (age) Men (M), Women (W)				Rating A B C	
Exposure/Intervention	Outcome variable	Study		Total	Animal	Vegetable		Strength of evidence: Convincing, probable, Suggestive, no conclusion
Frequency of protein consumption (per day/per week)	BMI	Cohort (53)	116 (18–31 years) M and W	NS	NA	NA	C	No conclusion
Quartiles E% of animal and vegetable protein intake	BMI: risk of overweight obesity	Cohort (54)	1,730 (40–55 years) M	NA NA	POS POS	NS INVERSE	C	
High protein diet (HP): 1.9 g/kg BW (22 E%) vs.	BMI	Randomized controlled trial (59)	15 (18–36 years) M	NS	NA	NA	C	
Normal diet (NP): < 1.3 g/kg BW (15 E%) in 6 months								

High protein diet (HP): 1.9 g/kg BW (22 E%) vs.	Body weight change	Randomized controlled trial (59)	15 (18–36 years) M	INVERSE	NA	NA	C	No conclusion
Normal diet (NP): < 1.3 g/kg BW (15 E%) in 6 months								
Total, animal and vegetable protein in kcal/day and per 150 kcal/day increments (equal 37.5 g protein)	Change in body weight in gram per year	6 cohorts (56)	89,432 M and W	POS	POS	NS	B	
Protein E%	5-yr change in body weight	Cohort (57)	1,762 M and W	NS	NA	NA	B	
Protein intake in servings/day	Weight gain of > 10 lb, yes or no	Cohort (58)	336 W	NS	NA	NA	C	
15 E% protein vs. 30 E%	Body weight change	Controlled trial (52)	19 M and W	INVERSE	NA	NA	B	

E% of total, animal. and vegetable protein	5-y change in waist circumference	Cohort (55)	42,969 M and W	INVERSE	INVERSE	NS	B	No conclusion
Total, animal and vegetable protein in kcal/day and per 150 kcal/day increments (equal 37.5 g protein)	6.5-y-change in waist circumference	Cohort (56)	89,432 M and W	NS	NS	NS	B	

High protein diet (HP): 1.9 g/kg BW (22 E%) vs.	Body composition:	Randomized controlled trial (59)	15 (18–36 years) M				C	No conclusion
Normal diet (NP): < 1.3 g/kg BW (15 E%) in 6 months	FFM (kg) FM (kg)			NS NS	NA NA	NA NA		

Regarding the evidence of a relationship between protein intake and changes in BW, three prospective cohort studies and two controlled trials are used. Halkjaer et al. (56) used country-specific FFQs from 89,432 men and women from six EPIC cohorts that were also included in the Diogenes project. After 6.5 years, they found weight gain to be significantly positively associated with total and animal protein intake. In the Danish Glostrup Population Studies and MONICA1 (57), the focus was on the energy density and fiber in relation to 5-year BW changes in adult men and women, but they also found a statistically non-significant positive association with the protein E%, assessed via weighed 7-day food records. Among US women consisting of 51% Caucasians, Sammel et al. (58) used a very simple FFQ with protein intake as ‘servings per day’. They found no statistically significant association with 4-year BW gain of ≥5 kg. In Ferrara et al.'s small RCT (59), 15 physically active men were prescribed an HP diet: 1.9 g/kg BW (22 E%) versus a normal diet (NP): <1.3 g /kg BW (15 E%). After 6 months, they found a significant decrease in BW of 2 kg in HP (3.5% of baseline BW) versus 0.7 kg in the NP group (1% of baseline BW). In another small but strictly controlled intervention study in 19 weight-stable men and women (52), the participants were on a weight-maintaining diet (15 E% protein, 35 E% fat, 50 E% CH) for 2 weeks, an isocaloric diet (30 E% protein, 20 E% fat, 50 E% CH) for 2 weeks and then an *ad libitum* diet (30 E% protein, 20 E% fat, 50 E% CH) for 12 weeks. During the *ad libitum* diet, the BW loss was 4.9±0.5 kg.

The evidence is assessed as *inconclusive* regarding the relation of protein intake to change in BW (Table 7).

Regarding the evaluation of the evidence of an association between protein intake and changes in waist circumference (WC), two prospective cohort studies are included. In the Danish Diet, Cancer and Health Study (55), where they used a validated FFQ designed for the study, the 5-year change in WC was inversely associated with E% of total and animal protein intake, while the Diogenes project (56) found no association between protein E% and a 6.5-year change in WC, based on country-specific FFQs.

The evidence is assessed as *inconclusive* regarding the relation of protein intake to change in WC (Table 7).

One RCT included body composition as the outcome. In Ferrara et al.'s small RCT (59) where 15 physically active men were prescribed an HP diet: 1.9 g/kg BW per day (22 E%) versus a normal diet (NP): 1.3 g/kg BW per day (15 E%) for 6 months, no association between protein intake and change in fat mass or FFM was found.

The evidence is assessed as *inconclusive* regarding the relation of protein intake to change in body composition (Table 7).

### Renal function and kidney stones

The evaluation of the association between protein and renal function based on GFR is based on two prospective cohort studies (11, 60), both quality graded as C, and two short-term intervention studies (9, 61), both quality graded as B. The short-term studies were included because of the HP content in the intervention diet (see Appendix C, Table C13). An association between protein intake and renal function may be explained by the increase in GFR observed after an increase in protein intake, which in the long-term may lead to increased glomerular pressure (62).

In a 7-year prospective cohort study among healthy individuals, Halbesma et al. (11) found no association between protein intake and decline in GFR, as estimated from plasma creatinine (eGFR). The cohort was separated into two groups according to 24-h urinary albumin ≥ or<10 mg/day. In the Nurses’ Health Study with 11-year follow-up among healthy women, Knight et al. (60) also found no association between protein intake and decline in eGFR in participants with a normal eGFR at baseline. Among women with mild kidney insufficiency at baseline, the decline in GFR was related to protein intake, significant also for non-dairy protein intake. The protein intake was based on an FFQ, and there was no information about the total energy intake. The cross-over study by Frank et al. (61) showed an increase in GFR with HP intake among young healthy male participants. This was confirmed by Walrand et al. (9) who studied both sexes of young participants. In contrast, Walrand found no increase in GFR among healthy elderly of both sexes.

The evidence is assessed as *inconclusive* regarding the relation of protein to renal function based on GFR (Table 8).

**Table 8:** Summary renal function and kidney stones

				Association of protein/effect (in RCT)				
			Number of participants (age) Men (M), Women (W)				Rating A B C	
Exposure/Intervention	Outcome variable	Study		Total	Animal	Vegetable		Strength of evidence: Convincing, probable, Suggestive, no conclusion
Experimental normal (1.2 g/kg per day) or high protein intake (2.4 g/kg per day)	GFR	RC cross-over intervention study (61)	24 men, average age 24 years	POS	POS	NA	B	No conclusion
Experimental normal (1 ≈ g/kg per day) or high protein intake (≈ 2 g/kg per day) in young and elderly.	GFR	Balance study (9)	10 young (24 years), 10 elderly (70 years), 5 women in each group	POS (young) NS (elderly)	NA	NA	B	
Quintiles of estimated protein intake (24-h N)	eGFR	Cohort (11)	6,000 with 24 h urinary albumin ≥ 10 mg/L. 2,592 with 24 h urinary albumin < 10 mg/L Average age: 50.	NS	NA	NA	C	
Protein intake (FFQ) in gram per day and in quintiles	eGFR	Cohort (60)	1,624 W	NS INVERSE in women with mild kidney insufficiency at baseline	NA	NA	C	

Experimental normal (1.2 g/kg per day) or high protein intake (2.4 g/kg per day)	Microalbuminuria	Experimental study (61)	24 men, average age: 24 years	POS	POS	NA	B	No conclusion
Experimental normal (1.5 g/kg per day) or high protein intake (3.0 g/kg per day)	Microalbuminuria	Experimental study (63)	24 men, average age: 24 years	NS	NA	NA	A	
Quintiles of estimated protein intake (24 h N)	Microalbuminuria	Cohort (11)	6,000 with 24 h urinary albumin ≥ 10 mg/L. 2,592 with 24 h urinary albumin < 10 mg/L Average age: 50	NS	NA	NA	C	
Protein intake (FFQ) in gram per day and in quintiles)	Microalbuminuria	Cohort (60)	1,624 W	NS	NA	NA	C	

Spontaneous intake (FFQ) energy-adjusted gram per day and quintiles	Kidney stone	Cohort (64)	96,245 W (27–44 years (average: 36 years)	NA	NS	NA	C	No conclusion
Spontaneous intake (FFQ) energy-adjusted in quintiles	Kidney stone	Cohort (65)	45,619 M. Average age not given, range of age groups: 40–≥ 70 years	NA	POS (Increase in group BMI <25, not overall)	NA	C	

The evaluation of the association between protein and renal function based on microalbuminuria is investigated in the same studies as GFR, namely two prospective cohort studies (11, 60), both quality graded as C, and one intervention study (61), quality graded as B, but also in a short-term study by Jakobsen et al. (63), quality graded as A (see Appendix C, Table C13).

The two cohort studies (11, 60) found no association between protein intake and urinary albumin excretion. The experimental cross-over study (61) among young healthy male volunteers showed that 7 days of HP intake (2.4 g/kg BW per day) considerably increased urinary albumin excretion (from 9 to 18 mg/day), as compared to a control protein intake of 1.2 g/kg BW per day. There were no changes in renal blood flow, renal vascular resistance, BP, or plasma levels of renin, aldosterone, or angiotensin II. The 3-week study of a similar increase in protein intake in young males (63) found no increase in urinary albumin excretion.

The evidence is assessed as *inconclusive* regarding the relation of protein to renal function based on microalbuminuria (Table 8).

The evaluation of the association between protein and risk of kidney stones is based on two 8- or 10-year prospective cohort studies (64, 65), both quality graded as C (see Appendix C, Table C14). Overall, there was no association between protein intake and kidney stone formation. One of the studies (65) found a higher risk with increased animal protein intake among men with a BMI<25, but no explanation could be offered for this observation. Thus, it cannot be entirely ruled out that an HP intake may promote kidney stone formation in normal weight men, but this suggestion is weakened by the low quality of the study.

The evidence is assessed as *inconclusive* regarding the relation of protein to risk of kidney stones (Table 8).

### Diabetes and glucose control

The evaluation of the association between protein intake and the onset of T2D is based on four prospective cohort studies (66–69), all quality graded as B (see Appendix C, Table C15). An association may be explained by the effect of amino acids on insulin sensitivity (68).

In the Health Professionals’ Follow-up Study of middle-aged men followed for 20 years (66), a LC-high total protein and fat score based on E% was associated with an increased risk of T2D, and the risk was even higher when the score was based on animal sources, mainly red and processed meat. The lowest quintile of total protein intake was 15.7 E% and the highest quintile 21.5 E%. The Nurses’ Health Study of middle-aged women followed for 20 years (67) found no association between an LC-high total protein and fat score and risk of TD2, except for a decreased risk when the score was based on vegetable sources. The lowest quintile of total protein intake was 14.7 E% and the highest quintile was 18.4 E%. The EPIC-Potsdam Study (68) in men and women found a decreased risk of T2D for each isoenergetic 5 E% higher contribution by CHs at the expense of protein, i.e. an increased risk related to an LCHP diet. Also, the EPIC-NL study among men and women (69) found an increased risk of T2D with increasing protein intake per 10 gram and with an isoenergetic substitution of 5 E% protein with CHs, resulting in an LCHP diet, and also when the protein intake was based on animal sources.

All of the diet results were obtained by FFQs.

Only one small study (70), quality graded as C, addressed the association between protein intake and blood glucose (see Appendix C, Table C15) and thus no conclusion can be drawn.

The evidence is assessed as *suggestive* regarding the relation of total and animal protein intake to increased risk of T2D, based on long-term LCHP diets, including one study with an LCHP-high-fat diet, while the evidence is assessed as *inconclusive* regarding the relation of total protein to fasting blood glucose (Table 9).

**Table 9:** Summary diabetes

				Association of protein/effect (in RCT)				
			Number of participants (age) Men (M), women (W)				Rating A B C	
Exposure/Intervention	Outcome variable	Study		Total	Animal	Vegetable		Strength of evidence: Convincing, probable, Suggestive, no conclusion
12 weeks on recommended protein (RP) ‘15 E% protein, 30 E% fat, 55 E% carbohydrate’ or high protein (HP) ‘25 E% protein, 30 E% fat and 45 E% carbohydrate’.	Fasting blood glucose	Intervention study (70)	Age ≈ 20 years				C	No conclusion
Groups:								
Body fat <30% of body weight.			N =34 in RP and 15 in HP	INVERSE	NA	NA		
Body fat ≥30% of body weight.			N =38 in RP and 7 in HP	NS	NA	NA		
Both groups also instructed to reduce usual energy intake by 500 kcal/d								

Quintiles of a low carbohydrate/high protein and fat score, and also based on animal or vegetable sources	New type 2 diabetes (T2D)	Cohort study (66)	40,475 M (40–75 years)	POS	POS	NS	B	Suggestive evidence that a low carbohydrate-high protein diet based on total and animal protein increases risk of T2D
Deciles of a low carbohydrate/ high protein and fat score, and also based on animal or vegetable sources		Cohort study (67)	85,059 W (30–55 years)	NS	NS	INVERSE	B	
Protein E% intake, substituted isoenergetically by 5 E% lower carbohydrate intake		Cohort study (68)	9,702 M (40–65 years) and 15,365 W (35–65 years)	POS	NA	NS	B	
Protein intake:		Cohort study (69)	2 cohorts mixed 38,094				B	
1) per 10 gram of intake and			M and W (age groups from	POS	POS	NS		
2) Quartiles of protein E% intake substituted isoenergetically by 5 E% lower carbohydrate intake			21 to 79 years)	POS	NS	NS		

### Physical training

The evaluation of the impact of physical training on protein requirement is based on three clinical trials (71–73), quality graded as C, B, and B, respectively (see Appendix C, Table C16). Increased protein use for building and repair of muscle tissue in periods of strength training, together with an increase in protein oxidation during endurance training, have been suggested as potential mechanisms underlying an association between training status and protein requirement.

The effect of aerobic exercise training on whole-body protein turnover during a set level of protein intake was tested in a study of seven young men and women (pooled) using stable isotope methodology (71). Protein intake was adjusted to 0.88 g/kg BW per day during a 2-week adaptation to the study diet. Thereafter, the subjects participated in 4 weeks of endurance training (walking and running 4–5 times per week at 85% of maximal heart rate), while following the study diet. The data indicated improved protein utilization in response to the exercise training; improved N-balance, decreased protein oxidation, and a tendency toward an improvement in non-oxidative leucine deposition (measurement of whole-body protein synthesis). The study may be underpowered for the rate of appearance of leucine (measurement of whole-body protein breakdown). No non-exercise control group was included, and only one level of protein intake was studied. For N-balance, no measurement was carried out on the completeness of urine collection.

A longer training study was performed by Hartman et al. (72) who studied the response to 12 weeks of resistance exercise training (whole-body split routine five times/week) in eight young men. Whole-body nitrogen flux (Q), protein synthesis (PS), protein breakdown (PB), and net protein balance (NPB=PS-PB) was measured by a stable isotope tracer of glycine before and after the exercise program during a 5-day period with controlled macronutrient intake (1.2 g protein/kg BW/day). Reductions were found in both PS and PB after the training program, whereas the net balance between synthesis and breakdown improved, suggesting that dietary requirements for protein in resistance trained formerly novice athletes, are not higher, but rather lower after resistance training.

Thalacker-Mercer et al. (73) analyzed 4-day dietary records on 60 participants previously clustered (K-means cluster analysis) as non-, modest-, and extreme-responders to 16 weeks of high-intensity resistance training (3-day/week), based on the magnitudes of change in m. vastus lateralis myofiber cross-sectional area. Despite marked variations in responses in the different groups, no differences were found among clusters in daily intake of protein or other macronutrients. The authors concluded that the observed protein intakes (ca. 1.1 g/kg BW/day in modest and extreme) were sufficient to facilitate modest and extreme muscle growth during resistance training. There may have been under-reporting of energy intake in all clusters, based on the reported intake relative to BW.

The evidence is assessed as *suggestive* for the effect of training on whole-body protein retention, but *inconclusive* regarding the effect of physical training on protein requirements (Table 10).

**Table 10:** Summary physical training

Exposure/Intervention	Outcome variable	Study	Number of participants (age) M, W	Effect of training/protein	Rating A B C	Strength of evidence: Convincing, probable, Suggestive, no conclusion
2 week dietary adjustment period (0.88 g protein/kg BW/day followed by 4 week progressive aerobic exercise training program while on the diet	Dietary requirement of protein expressed via: whole body protein turnover/Protein retention	Clinical trial (71)	8 (18–25 years), 3M, 4W	Improved N-balance and protein utilization	C	Suggestive for effect of physical training No conclusion regarding dietary requirement
Whole body split resistance training, 12 weeks, 5 days/week Diet 15 E% protein. 5-day periods pre- and post with controlled diets (1.2 g/kg BW/day).	Whole-body protein turnover/Protein retention	Clinical trial (72)	8 (22+1 years), 8M	Improved N-balance and protein utilization	B	
16 wk progressive resistance training, 3-day/ week, lower and upper body exercises, hard intensities. Protein intake app. 1 g/kg BW/day	Muscle hypertrophy/‘Protein retention’	Clinical trial (73)	60 [20–35 years (18M and 14W) and 60–75 years (14M and 14W)], 32M, 28W	Protein intake sufficient to induce muscle hypertrophy/‘Protein retention’	B	

## Discussion

The main findings of this systematic review on protein intake and the relation to health outcomes in healthy adult populations comparable to the Nordic populations are that the evidence is assessed *probable* regarding the estimated average requirement based on N-balance studies, while an estimation of an optimal level of protein intake based on the evidence of the relationships of protein intake to mortality and morbidity are ranging from *suggestive* to *inconclusive*.

It should be noted that the grading of the evidence was only based on studies from 2000 to 2011 and for some outcomes, and the inclusion of earlier studies might have resulted in different grading. On the other hand, the most recent recommendations of protein intake were also based on N-balance studies, while the relation to health outcomes was considered insufficient to establish reference values (5) or recommendations (4). Studies with total, animal, or vegetable protein were included in this review, while studies at amino acid level were not included. The usual diet in Nordic countries is considered unlikely to be limited in their content of indispensable amino acids, and thus, we did not regard it relevant to make an update of the comprehensive work by WHO/FAO/UNU expert group from 2007 about amino acid requirements (4).

We only included studies with healthy adults, primarily long-term studies under free-living conditions. Postprandial (acute/single-meal studies) and short-term studies may not reflect the effect from *ad libitum* long-term dietary habits and/or mechanisms such as adaptation. Studies including both healthy persons and persons with risk factors show different outcomes on nutrition exposures.

Despite limitations in the method mainly related to accuracy of the measurements and interpretation of the results, N-balance remains the method of choice for determining protein requirement in adults in the absence of validated or accepted alternatives and in the absence of a reliable biological marker of protein status. Rand et al.'s meta-analysis (3) included N-balance studies starting with those cited in the 1985 FAO/WHO/UNU report (74) and supplemented with an electronic search in MEDLINE. They found no statistically significant differences between climate of study site, adult age, class, sex, and source of dietary protein, although there was an indication that women might have a lower requirement. The authors underlined that the data did not provide sufficient power to detect possible differences. It is also noteworthy that only one study with elderly persons was included. In our review, we included two additional balance studies published after the meta-analysis. The high-quality-graded N-balance study by Campbell et al. (8) found no difference in the estimated requirement between young and old participants or men versus women, while a small study (9) found that the net daily N-balance increased equally in the younger and older participants on an HP diet (22–24 E%). From all of these studies, the evidence is assessed as *probable* regarding a median-estimated average requirement of nitrogen of 105 mg/kg BW per day corresponding to 0.66 g good-quality protein/kg BW per day, regardless of sex and age.

The use of nitrogen balance to establish protein dietary recommendations can be discussed. This methodology is an indirect determination of protein turnover, and no information about whole-body nitrogen or protein turnover, or various protein metabolic pathways, can be obtained. Furthermove, achievement of complete urine collections and strict measurements of energy intake/balance is challenging in field studies. If the energy balance changes during the study, this will influence the results. Also, low protein intake may induce protein sparing, and thus lead to underestimation of needs. As evidenced by our results, there is a lack of rigorously controlled long-term studies based on nitrogen balance. During the last decades, more direct methods to measure turnover of various body proteins have been applied, including stable isotope tracer methodology. This has enabled a mechanistic approach to the effects of various dietary proteins. However, the main limitation to date is the lack of prolonged studies using this methodology. This is evidenced by the fact that most articles using stable isotope methodology in our search from 2000 and onward describe only acute effects of protein or amino acid intake (see Appendix B) and mainly on muscle protein metabolism. However, WHO/FAO/UNU (4) used stable isotope studies to increase the requirements for essential amino acids based on the biologically sound criterion that the point of intake where oxidation of the essential amino acids investigated begins to increase, reflects the point of intake above requirements. Similar logical reasoning is not available for whole-body protein turnover beyond what can alreadyy be deduced from N-balance studies. Rates of whole-body protein synthesis and degradation are usually reported to increase in parallel with protein intakes above the amount required for N-balance, but the relationship between whole-body protein turnover rate and health or body functions, needs to be established. Studies of turnover of muscle proteins have similarly not yet added to an understanding of muscle function, since no studies are available demonstrating a correlation between, e.g. muscle strength or endurance and the dynamics of muscle protein turnover. Thus, it will be important to use more advanced methodology in future strictly controlled long-term studies in order to establish mechanistic links between protein intake from various sources and health outcomes.

The relationship between total protein intake and all-cause mortality is regarded as *inconclusive*. Only the PREVEND study included protein intake *per se* (11), and they used baseline protein intake estimated from two collections of 24-h urinary nitrogen with no correction for possible loss of urine, and the intake expressed as g protein per kg ‘ideal’ BW (after correcting the BMI to 22). Thus, the estimated mean intake of 1.20 g protein/kg ideal BW was probably higher than the actual intake. They found an inverse relation to all-cause mortality. The Iowa Women's Health Study (12) used baseline protein E% in quintiles (substituted isoenergetically for CHs) estimated from an FFQ, with no information about the estimated energy intake, and they found no relation to all-cause mortality. The remaining three studies (10, 13, 16) with four cohorts including ca. 200,000 men and women, used an LCHP diet score based on the protein E%, and also an LCHP and high-fat score (10), and found an increased risk of all-cause mortality. Thus, we regard the evidence as *suggestive* for a relationship between diet with an LCHP score and increased risk of all-cause mortality, but the use of an LCHP score makes it uncertain whether the effects result from reduced CH or increased protein and/or fat. It is also noteworthy that the authors emphasized that the data evaluated the health consequences of long-term habitual dietary intakes and should not be interpreted as indicating that short-term use of LCHP diets is detrimental to health. Regarding cardiovascular mortality and total intake of protein *per se*, the evidence is assessed as *inconclusive* based on LCHP scores in two studies that found positive associations (13, 16), one study with a non-significant association (10), and based on protein E% both non-significant (12) and inverse (15) relations. In the three studies that included the sources of protein (10, 12, 14) quality assessed as B, C, and B, respectively, an inverse relation to cardiovascular mortality was found in all studies. Thus, we assess the evidence as *suggestive* for a protective effect of vegetable protein, including an LCHP diet based on vegetable protein, toward cardiovascular mortality. No statistically significant associations between total protein intake or LCHP diets and cancer mortality was found, and the only study that also included protein sources found non-significant relations to animal- and vegetable-based protein (12). Most studies on the relationship between protein intake and cancer are food-based (6), and therefore cannot isolate the protein effect *per se*.

The overall association between morbidity and intake of total protein is assessed as *inconclusive* and thus in agreement with other recent reviews (e.g. (4, 5)).

The relation to breast cancer is regarded is *inconclusive*, based on The Nurses’ Health study (17) where no significant association was found to either energy-adjusted total or animal–vegetable-based protein intake, and on one case-control study that found an increased risk in the highest tertile of total protein intake in gram per day (18), while another small case-control study found no relation to energy-adjusted total, animal- or vegetable-based protein (19). None of these studies included heredity as a confounder. The relation to colorectal cancer is regarded as *inconclusive*, based on a meta-analysis, primarily including studies with foods and with main focus on animal sources of fat and protein (20), and on three case-control studies (21–23) that found no significant associations. It is noteworthy that in a Norwegian study, the non-significant differences were most marked when healthy controls were used as the comparison group (21), thus, drawing attention to the use of hospital controls versus healthy controls. Regarding the relation to other cancers (laryngeal, Non-Hodgkin's lymphoma, esophageal and gastric cancer, ovarian, pancreatic and prostate cancer), the evidence is based on only one case-control study for each outcome and thus inconclusive, while the non-significant association with renal cell cancer was based on a pooled analysis of 13 prospective cohort studies (30). However, there was no information about the intake level in the quintiles of protein intake used in the analysis.

For cardiovascular diseases, the association between protein intake and coronary heart disease and strokes was statistically non-significant in the six included cohort studies and thus regarded as *inconclusive*. However, it is noteworthy that the Health Professionals’ Follow-Up Study (33) showed a statistically significant relationship between increased risk of IHD and total and animal protein expressed as quintiles of E% substituted for an isocaloric amount of CHs, but only in the subgroup of ‘healthy’ men (without baseline hypertension, diabetes, and hypercholesterolemia). The association between protein intake and BP is regarded as *inconclusive* for total and animal protein, while the association with vegetable protein intake is regarded as *suggestive*. Thus, we are in agreement with the WHO/FAO/UNU report (4) that concluded ‘This is an obvious area for further research aimed at identifying causality and, if causality exists, determining whether the effect is attributable to proteins of plant or animal origin’. We included one feeding study (34), The OmniHeart study that was based on a CH diet similar to the Dietary Approaches to Stop Hypertension (DASH) diet, but with 15 E% protein versus 25 E% protein, and the 10 E% protein replaced with CHs. The subgroup analysis in the 40% Caucasians found no significant relationship between protein intake and BP. DASH studies were not included in our review because they were based on a feeding study that tested the effects of dietary patterns rather than individual nutrients and was thus not designed to identify the specific nutrients and foods responsible for the observed reductions in BP (75). Both the SUN cohort study (35) and the Chicago Western Electric Study (36) found an inverse relation of E% of vegetable protein and risk of hypertension/BP, and the most recent meta-analysis with soya intake in controlled trials support that the evidence is regarded as *suggestive*. However, it is still difficult to separate the effect of vegetable protein from the other nutrients in vegetables (e.g. potassium, fiber) that influence BP. Two good-quality meta-analyses of randomized controlled studies (39, 40) found a statistically significant inverse effect of a mean daily intake of 25–30 g soya protein, corresponding to 1–2 servings per day, on LDL cholesterol. Studies with highest baseline LDL had greater reductions than studies with the lowest values, thus, the effect may be smaller in normo-cholesterolemic subjects. The evidence is assessed as *probable to convincing* regarding the effect of soya protein on LDL cholesterol, but the intake levels are much higher than in the present Nordic diet and thus questions the relevance in the average Nordic diet.

The role of dietary protein on bone health has been controversial. On the one hand, urinary calcium loss is increased by HP intakes, while, on the other hand, protein increases calcium absorption or bioavailability, which questions the net effect of HP diets on calcium economy and the effect on bone health (76). Any negative effect of protein may also be opposed by an increase in the protein-sensitive insulin-like growth factor 1, IGF-1. We assess the evidence as *inconclusive* regarding the relation of protein intake to an overall effect on calcium and bone metabolism and is thus in line with EFSA (5) that found the available evidence regarding protein and bone health to be insufficient. Protein intake and risk of bone loss was based on three small cohort studies (41–43), all quality graded C, mainly with women, and a good-quality meta-analysis (44) that found a ‘small benefit of protein on bone health’, but the overall association is *inconclusive* regarding benefit or adverse effects of higher protein intake. According to the meta-analysis (44) and the three cohort studies that included risk of fractures (42, 45, 46), the association with protein intake is *inconclusive*, but there seemed to be an (inconclusive) interaction with the intake level of calcium. Fenton et al. (47) made a fine systematic review and meta-analysis on the association between dietary acid load, including protein intake, and bone health, but unfortunately the information about the dietary intakes/interventions was insufficient, and thus quality was assessed as C. They concluded that ‘The analysis did not find support for the hypothesis that “acid” from the diet causes osteoporosis or that an “alkaline” diet prevents osteoporosis. Higher protein intakes and animal protein were not detrimental to calcium retention. The ideal protein intake for bone health could not be determined.’ The two good-quality intervention trials that addressed calcium and bone metabolism used a combination of sodium and protein effects (48) or protein and calcium (49), and thus, the effect of protein *per se* could not be assessed.

Regarding the association between protein and energy intake, BW control and body composition, we excluded studies with overweight/obese participants, or participants on weight loss diets since factors involved in weight loss among obese may differ from factors responsible for weight gain in the normal weight (57). Overall, we found the associations *inconclusive*. EFSA (5) based their assessment on studies with mainly overweight/obese participants and concluded that ‘these studies are difficult to interpret with respect to whether the effects observed are due to an increase in dietary protein intake or to the concomitant modification of carbohydrate and/or fat intakes, and whether any observed effect of an increase in dietary protein would be sustainable’. Protein in relation to energy intake was examined in two intervention studies, where Rumpler et al. (51) found no effect of the three macronutrients in a small study, while Weigle et al. (52) found a decreased appetite during a combination of an HP-low-fat diet. The cohort study by Koppes et al. (50) found the association between protein and energy intake to be three times larger than the association between fat and energy intake. Thus, no conclusion could be drawn from the included studies. Regarding the association between protein and BMI, the included studies were generally of low quality, mainly because of insufficient dietary recording method (53) or description (54, 59). Two controlled trials found increased protein intake to induce weight loss; a small low-quality study (59) that found a 2 kg weight reduction ((3.5% of baseline BW) during 6 months, and Weigle et al.'s (52) small but high quality study with an HP-low-fat diet resulting in a weight loss of 4.9±0.5 kg (body fat 3.7±0.4 kg) after 12 weeks *ad libitum* diet. In observational studies, the EPIC study (56) found that total and animal protein increased the risk of weight gain in 89,000 men and women during a mean follow-up of 6.5 years, while two small cohort studies (57, 58) found non-significant associations. A comprehensive review (77) of the epidemiological evidence on the associations between diet and subsequent weight gain and obesity concluded: ‘The substantial evidence reviewed suggests that levels of protein intake, regardless of source, are not associated with subsequent excess weight gain or obesity, although the results were inconsistent.’ The conclusion was based on 11 prospective cohort studies from 1990 to 2007 of which we have included three studies (53, 57, 58) in our review. Regarding protein intake and the association with changes in WC, the results from two large prospective cohort studies were conflicting, because the Danish study with ca. 42,000 participants (55) found an inverse relation to total and animal protein intake, while the EPIC study (56) did not find significant association. Only one small RCT addressed changes in body composition (59), and it did not find that HP diet had any effect on body composition. This is a particular area that warrants further investigation. Weight loss in healthy adults, possibly induced by increased protein intake, is probably not associated with health benefits, but increased FFM may be of benefit.

Regarding the associations between protein kidney function and kidney stones, the evidence is regarded as *inconclusive*. EFSA also found the available evidence insufficient to derive an upper level of protein intake based on protein and kidney function (5). But our systematic review calls for reflection. The two included experimental studies (9, 61) found that an HP intake corresponding to ca. 2 g/kg BW compared to ca. 1 g/kg BW increased GFR among young participants, but this association was not confirmed by the two cohort studies (11, 60). The increase in GFR is a normal physiological adaption to increased protein intake (78) but it is also an important component of the hyperfiltration theory of Brenner (79) due to its presumed effect of increasing glomerular pressure. Walrand et al. (9) found that an HP intake did not increase GFR in the elderly participants, from a baseline GFR which was lower than that of the young participants. This is probably due to the reduced kidney function in elderly, since patients with mild-to-moderate chronic kidney disease also do not show the UP-induced increase in GFR (80). It is also an important component of the hyperfiltration theory that overloading of remnant nephron mass should be avoided. Caution is required due to the observation of a decline in GFR among women with mild kidney insufficiency (60). Regarding microalbuminuria, one experimental study found an increase in urinary albumin after 7 days on an HP intake of 2.4 g/kg BW per day (61), while a similar increase in protein intake in the other short-term experimental study of healthy young men did not find an increase in 24-h urinary albumin excretion (63). Further studies are needed to settle whether this discrepancy is due to the different durations of the studies or e.g. due to different methods of analysis of albumin in the urine. A review by Friedman (62) cites an earlier 3-week study showing a reduction in proteinuria with reduced protein intake (from 75 to 43 g/day). Caution is required until this is settled. Among patients with chronic kidney disease, the presence of albuminuria, even within the normal range, is a strong predictor of future decline in kidney function which is understood in the context of the hyperfiltration theory by Brenner (79).

The evidence is regarded as *suggestive* of an association between long-term LCHP diets and an increased risk of T2D based on three (66, 68, 69) out of four prospective cohort studies. In two of the studies (66, 69), this association was most clearly associated with intake of animal protein, possibly a reflection of the fact that animal protein was the main protein source. The association was absent if related only to the intake of vegetable protein (66, 69). However, as is clear from one study (69), the intake of vegetable protein was much lower than that of animal protein, which leaves it an open question whether the same result would be found with a higher intake of vegetable protein, covering the protein requirements. The study in women adjusted only for age, smoking, physical activity, alcohol, family history of T2D, BMI, and hormone use (67). The study of males only also adjusted for total energy and coffee (66). The two studies of men and women together (68, 69) did adjust for sex and, in addition to the confounders mentioned above, also adjusted for education, fiber intake, magnesium intake (68) and further, for WC, energy-adjusted intake of saturated fat, monounsaturated fat, polyunsaturated fat, cholesterol, vitamin E, glycemic load, and BP (69). The employment of different confounders for statistical adjustment may contribute to different results in such studies. However, the results from long-term LCHP diets make it uncertain whether the effects result from reduced CH or increased protein.

The association of physical training on protein retention is evaluated as *suggestive* based on two smaller studies using stable isotope technology (71, 72). In the study by Gaine et al. (71), young untrained adults participated in 4 weeks of aerobic training while their protein intake was kept strictly on 0.88 g/kg BW per day. An improved N-balance was found with training, together with reduced leucine oxidation and a tendency to improved non-oxidative leucine deposition (estimate of protein synthesis). Thus, improved protein utilization and nitrogen retention was found in response to aerobic exercise training in weight-stable subjects. In a longer training study by Hartman et al. (72), young untrained adults participated in 12 weeks of resistance exercise training. Their protein intake was 15 E% during the training period. Whole-body protein turnover was determined during 5 days with controlled diets (1.2 g protein/kg BW per day) before and after the training period. In this study, nitrogen flux, and whole-body protein synthesis and breakdown were reduced as a result of the training, whereas net whole-body protein balance (synthesis breakdown) and urinary N-balance improved. Thus, the study supports that exercise training increases protein retention, and also suggests that after longer training periods, that the protein requirement is reduced rather than increased. The response to exercise training may however vary between individuals, and in a study by Thalacker-Mercer et al. (73), no correlation was found between regular protein intake (or other macronutrient or energy intake) and leg muscle hypertrophic response to 16 weeks of resistance training, despite a variation of 0–60% hypertrophy between non- and extreme-responders. The average protein intake varied from 0.97 to 1.07 g/kg BW per day, and did not differ between groups of non-, moderate, and extreme responders. Thus, this protein intake was sufficient to support a large range of responses, and the lack of response to training could not be explained by a sub-optimal diet. However, the data indicate that there may have been substantial underreporting of energy intake in all three clusters.

The evidence is evaluated as *inconclusive* regarding the effect of physical training on protein requirements. In all three included studies, protein intake was on or above the RDA for healthy adults. It is unclear if the results would differ if protein intake was below a sufficient level to support muscle growth.

The literature search of interaction between physical activity and protein intake resulted, for the most part, in studies of short duration, studies in athletes, or studies of specific protein or amino acid supplements. Therefore, these were not included in the review. There is a lack of prolonged studies of the impact of (moderate) physical activity on dietary protein effects on various outcomes.

In summary, whereas exercise training may improve protein retention, the response may vary, and this cannot be explained by diet or protein intake alone. Our evaluation is in agreement with the most recent position statement on ‘Nutrition and Athletic Performance’ from the American Dietetic Association, Dietitians of Canada, and the American College of Sports Medicine (81), that states that protein requirement, even in athletes, can generally be achieved through diet alone without the use of protein or amino acid supplements. Thus, the same should be valid for healthy adults who are physically active. They do however recommend a slightly higher protein intake in endurance and strength-trained athletes with an intake ranging from 1.2 to 1.7 g/kg BW per day.

Overall for our systematic review, many of the protein intake data from the included observational studies are based on semi-quantitative FFQs, mainly from the large US prospective cohort studies, Nurses’ Health Study and the Health Professionals’ Follow-up Study, but also from the Women Health Initiative Observational Study and the EPIC studies. In some studies, the FFQs were ‘calibrated’ to correct for under- or over-reporting, and in other studies analyzed as ‘scores’ of intake of macronutrients making it difficult to separate the effect of protein *per se*. Many of the studies removed implausible energy intakes, but the range of the included intake remained large (e.g. including 2.5–16 MJ). None of the included studies used cut-off values for the energy intake: basal metabolic rate to assess the misreporting (82). The semi-quantitative FFQ have the ability to rank the subjects based on their intakes, so that subjects with low intakes can be separated from those with high intakes. This permits the calculation of the odds ratio or relative risk of disease in relation to intake (83). Based on calibrated data from the Women Health Initiative Observational Study about protein intake and the risk of frailty (84), it has been argued that FFQs better assess nutrient consumption as a fraction of total energy intake than absolute nutrient consumption. Thus, it may be more appropriate to conclude from protein E% than protein intake expressed in gram per day or in g/kg BW per day.

The main impression from our systematic review is that we agree with the conclusions from WHO/FAO/UNU 2007 (4):

- requirement is the lowest level of dietary protein intake that will balance the losses of nitrogen from the body, and thus maintain the body protein mass …
- … nitrogen balance does not necessarily identify the optimal intake for health, which is less quantifiable.
- There is emerging information on the apparently beneficial effect of protein intakes in excess of the safe level for lowering BP, reducing risk of ischemic heart disease and improving bone health. It is clearly urgent to identify whether such associations are causal, what the mechanisms are, and what the dose response is.
- The task is to identify protein intakes that enable long-term health and well-being.

Our systematic literature review has unfortunately failed to identify high-quality studies which could alter the classical criterion for protein recommendations, i.e. that requirement is the lowest level of dietary protein intake that will balance the losses of nitrogen from the body, and thus maintain the body protein mass.

## Conclusion

The evidence is assessed *probable* regarding the estimated average requirement based on N-balance studies.

The estimation of an optimal level of protein intake based on the evidence of the relations of total protein intake (regardless of source) to mortality and morbidity is mainly ranging from *suggestive* to *inconclusive*, i.e. *suggestive* for a relationship between increased all-cause mortality risk and long-term LCHP diets; *inconclusive* for a relationship between all-cause mortality risk and total protein intake *per se*; *suggestive* for an inverse relationship between cardiovascular mortality and vegetable protein intake; *inconclusive* for relationships between cancer mortality and cancer diseases, respectively, and protein intake; *inconclusive* for a relationship between cardiovascular diseases and total protein intake; *suggestive* for an inverse relationship between BP and vegetable protein; *probable* to *convincing* for an inverse relationship between soya protein intake and LDL cholesterol; *inconclusive* for a relationship between protein intake and bone health, energy intake, BW control, body composition, renal function, and risk of kidney stones, respectively; *suggestive* for a relationship between increased risk of T2D and long-term LCHP-high-fat diets; *inconclusive* for impact of physical training on protein requirement; and *suggestive* for effect of physical training on whole-body protein retention.

It is noteworthy, that many of the included studies found a decreased risk of outcome associated with vegetable protein intake.

Overall, many of the included prospective cohort studies were difficult to fully evaluate since results were mainly obtained by FFQs, which in some studies were ‘calibrated’ to correct for under- or over-reporting and in other studies analyzed as ‘scores’ of intake of macronutrients making it difficult to separate the effect from protein *per se*.

Regarding harmful effects of an HP intake, the evidence is regarded as *inconclusive*, but it cannot be entirely ruled out that an HP intake corresponding to ca. 24 E% or ca. 2 g/kg BW may affect kidney function in the long term. The evidence is assessed as *suggestive* regarding an increased risk of all-cause mortality and T2D in relation to long-term LCHP diets for a total protein intake of at least 20–23 E%, but the use of an LCHP score makes it uncertain whether the effects result from reduced carbohydrate or increased protein intake. Potentially adverse effects of a protein intake exceeding 20–23 E% remain to be investigated.

## References

[1] Nordic Nutrition Recommendations 2004 (2004). Integrating nutrition and physical activity.

[2] Institute of Medicine (2002). Dietary reference intakes for energy, carbohydrates, fiber, fat, protein and amino acids (Macronutrients).

[3] Rand WR, Pellett PL, Young VR (2003). Meta-analysis of nitrogen balance studies for estimating protein requirements in healthy adults. Am J Clin Nutr.

[4] WHO/FAO/UNU (2007). Protein and amino acids requirements in human nutrition: report of a joint WHO/FAO/UNU expert consultation.

[5] European Food safety Authority (EFSA) (2012). EFSA panel on dietetic products, nutrition and allergies (NDA); scientific opinion on dietary rerence values for protein. EFSA J.

[6] World Cancer Research Fund/American Institute for Cancer Research (2007). Food, nutrition, physical activity and the prevention of cancer: a global perspective.

[7] NNR5 Working Group (2011). A guide for conducting systematic literature reviews for the 5th edition of the Nordic nutrition recommendations.

[8] Campbell WW, Johnson CA, McCabe GP, Carnell NS (2008). Dietary protein requirements of younger and older adults. Am J Clin Nutr.

[9] Walrand S, Short KR, Bigelow ML, Sweatt AJ, Hutson SM, Nair KS (2008). Functional impact of high protein intake on healthy elderly people. Am J Physiol Endocrinol Metab.

[10] Fung TT, van Dam RM, Hankinson SE, Stampfer M, Willett WC, Hu FB (2010). Low-carbohydrate diets and all-cause and cause-specific mortality: two cohort studies. Ann Intern Med.

[11] Halbesma N, Bakker SJ, Jansen DF, Stolk RP, De Zeeuw D, De Jong PE (2009). High protein intake associates with cardiovascular events but not with loss of renal function. J Am Soc Nephrol.

[12] Kelemen LE, Kushi LH, Jacobs DR, Cerhan JR (2005). Associations of dietary protein with disease and mortality in a prospective study of postmenopausal women. Am J Epidemiol.

[13] Lagiou P, Sandin S, Weiderpass E, Lagiou A, Mucci L, Trichopoulos D (2007). Low carbohydrate-high protein diet and mortality in a cohort of Swedish women. J Intern Med.

[14] Preis SR, Stampfer MJ, Spiegelman D, Willett WC, Rimm EB (2010). Dietary protein and risk of ischemic heart disease in middle-aged men. Am J Clin Nutr.

[15] Prentice RL, Huang Y, Kuller LH, Tinker LF, Horn LV, Stefanick M (2011). Biomarker-calibrated energy and protein consumption and cardiovascular disease risk among postmenopausal women. Epidemiology.

[16] Trichopoulou A, Psaltopoulou T, Orfanos P, Hsieh CC, Trichopoulos D (2007). Low-carbohydrate-high-protein diet and long-term survival in a general population cohort. Eur J Clin Nutr.

[17] Holmes MD, Colditz GA, Hunter DJ, Hankinson SE, Rosner B, Speizer FE (2003). Meat, fish and egg intake and risk of breast cancer. Int J Cancer.

[18] Sala E, Warren R, Duffy S, Welch A, Luben R, Day N (2000). High risk mammographic parenchymal patterns and diet: a case-control study. Br J Cancer.

[19] Sieri S, Krogh V, Muti P, Micheli A, Pala V, Crosignani P (2002). Fat and protein intake and subsequent breast cancer risk in postmenopausal women. Nutr Cancer.

[20] Alexander DD, Cushing CA, Lowe KA, Sceurman B, Roberts MA (2009). Meta-analysis of animal fat or animal protein intake and colorectal cancer. Am J Clin Nutr.

[21] Almendingen K, Hofstad B, Trygg K, Hoff G, Hussain A, Vatn M (2001). Current diet and colorectal adenomas: a case-control study including different sets of traditionally chosen control groups. Eur J Cancer Prev.

[22] Breuer-Katschinski B, Nemes K, Marr A, Rump B, Leiendecker B, Breuer N (2001). Colorectal adenomas and diet: a case-control study. Dig Dis Sci.

[23] Levi F, Pasche C, Lucchini F, La Vecchia C (2002). Macronutrients and colorectal cancer: a Swiss case-control study. Ann Oncol.

[24] Bosetti C, La Vecchia C, Talamini R, Negri E, Levi F, Fryzek J (2003). Energy, macronutrients and laryngeal cancer risk. Ann Oncol.

[25] Zheng T, Holford TR, Leaderer B, Zhang Y, Zahm SH, Flynn S (2004). Diet and nutrient intakes and risk of non-Hodgkin's lymphoma in Connecticut women. Am J Epidemiol.

[26] Mayne ST, Risch HA, Dubrow R, Chow WH, Gammon MD, Vaughan TL (2001). Nutrient intake and risk of subtypes of esophageal and gastric cancer. Cancer Epidemiol Biomarkers Prev.

[27] Pan SY, Ugnat AM, Mao Y, Wen SW, Johnson KC, Canadian Cancer Registries Epidemiology Research Group (2004). A case-control study of diet and the risk of ovarian cancer. Cancer Epidemiol Biomarkers Prev.

[28] Lucenteforte E, Talamini R, Bosetti C, Polesel J, Franceschi S, Serraino D (2010). Macronutrients, fatty acids, cholesterol and pancreatic cancer. Eur J Cancer.

[29] Hu J, La Vecchia C, Gibbons L, Negri E, Mery L (2010). Nutrients and risk of prostate cancer. Nutr Cancer.

[30] Lee JE, Spiegelman D, Hunter DJ, Albanes D, Bernstein L, van den Brandt PA (2008). Fat, protein, and meat consumption and renal cell cancer risk: a pooled analysis of 13 prospective studies. J Natl Cancer Inst.

[31] Halton TL, Willett WC, Liu S, Manson JE, Albert CM, Rexrode K (2006). Low-carbohydrate-diet score and the risk of coronary heart disease in women. N Engl J Med.

[32] Iso H, Stampfer MJ, Manson JE, Rexrode K, Hu F, Hennekens CH (2001). Prospective study of fat and protein intake and risk of intraparenchymal hemorrhage in women. Circulation.

[33] Preis SR, Stampfer MJ, Spiegelman D, Willett WC, Rimm EB (2010). Lack of association between dietary protein intake and risk of stroke among middle-aged men. Am J Clin Nutr.

[34] Appel LJ, Sacks FM, Carey VJ, Obarzanek E, Swain JF, Miller ER (2005). Effects of protein, monounsaturated fat, and carbohydrate intake on blood pressure and serum lipids: results of the OmniHeart randomized trial. JAMA.

[35] Alonso A, Beunza JJ, Bes-Rastrollo M, Pajares RM, Martínez-González MA (2006). Vegetable protein and fiber from cereal are inversely associated with the risk of hypertension in a Spanish cohort. Arch Med Res.

[36] Stamler J, Liu K, Ruth KJ, Pryer J, Greenland P (2002). Eight-year blood pressure change in middle-aged men: relationship to multiple nutrients. Hypertension.

[37] Liu L, Ikeda K, Sullivan DH, Ling W, Yamori Y (2002). Epidemiological evidence of the association between dietary protein intake and blood pressure: a meta-analysis of published data. Hypertens Res.

[38] Dong JY, Tong X, Wu ZW, Xun PC, He K, Qin LQ (2011). Effect of soya protein on blood pressure: a meta-analysis of randomised controlled trials. Br J Nutr.

[39] Harland JI, Haffner TA (2008). Systematic review, meta-analysis and regression of randomised controlled trials reporting an association between an intake of circa 25 g soya protein per day and blood cholesterol. Atherosclerosis.

[40] Anderson JW, Bush HM (2011). Soy protein effects on serum lipoproteins: a quality assessment and meta-analysis of randomized, controlled studies. J Am Coll Nutr.

[41] Beasley JM, Ichikawa LE, Ange BA, Spangler L, LaCroix AZ, Ott SM (2010). Is protein intake associated with bone mineral density in young women?. Am J Clin Nutr.

[42] Sellmeyer DE, Stone KL, Sebastian A, Cummings SR (2001). A high ratio of dietary animal to vegetable protein increases the rate of bone loss and the risk of fracture in postmenopausal women. Study of Osteoporotic Fractures Research Group. Am J Clin Nutr.

[43] Tucker KL, Hannan MT, Kiel DP (2001). The acid-base hypothesis; diet and bone in the Framingham Osteoporosis Study. Eur J Nutr.

[44] Darling AL, Millward DJ, Torgerson DJ, Hewitt CE, Lanham-New SA (2009). Dietary protein and bone health: a systematic review and meta-analysis. Am J Clin Nutr.

[45] Dargent-Molina P, Sabia S, Touvier M, Kesse E, Bréart G, Clavel-Chapelon F (2008). Proteins, dietary acid load, and calcium and risk of postmenopausal fractures in the E3N French women prospective study. J Bone Miner Res.

[46] Sahni S, Cupples LA, McLean RR, Tucker KL, Broe KE, Kiel DP (2010). Protective effect of high protein and calcium intake on the risk of hip fracture in the Framingham offspring cohort. J Bone Miner Res.

[47] Fenton TR, Tough SC, Lyon AW, Eliasziw M, Hanley DA (2011). Causal assessment of dietary acid load and bone disease: a systematic review & meta-analysis applying Hill's epidemiologic criteria for causality. Nutr J.

[48] Harrington M, Bennett T, Jakobsen J, Ovesen L, Brot C, Flynn A (2004). The effect of a high-protein, high-sodium diet on calcium and bone metabolism in postmenopausal women and its interaction with vitamin D receptor genotype. Br J Nutr.

[49] Hunt JR, Johnson LK, Fariba Roughead ZK (2009). Dietary protein and calcium interact to influence calcium retention: a controlled feeding study. Am J Clin Nutr.

[50] Koppes LL, Boon N, Nooyens AC, van Mechelen W, Saris WH (2009). Macronutrient distribution over a period of 23 years in relation to energy intake and body fatness. Br J Nutr.

[51] Rumpler WV, Kramer M, Rhodes DG, Paul DR (2006). The impact of the covert manipulation of macronutrient intake on energy intake and the variability in daily food intake in nonobese men. Int J Obes.

[52] Weigle DS, Breen PA, Matthys CC, Callahan HS, Meeuws KE, Burden VR (2005). A high-protein diet induces sustained reductions in appetite, ad libitum caloric intake, and body weight despite compensatory changes in diurnal plasma leptin and ghrelin concentrations. Am J Clin Nutr.

[53] Adams T, Rini A (2007). Predicting 1-year change in body mass index among college students. Am Coll Health.

[54] Bujnowski D, Xun P, Daviglus ML, Van Horn L, He K, Stamler J (2011). Longitudinal association between animal and vegetable protein intake and obesity among men in the United States: the Chicago Western Electric Study. J Am Diet Assoc.

[55] Halkjaer J, Tjønneland A, Thomsen BL, Overvad K, Sørensen TI (2006). Intake of macronutrients as predictors of 5-y changes in waist circumference. Am J Clin Nutr.

[56] Halkjaer J, Olsen A, Overvad K, Jakobsen MU, Boeing H, Buijsse B (2011). Intake of total, animal and plant protein and subsequent changes in weight or waist circumference in European men and women: the Diogenes project. Int J Obes.

[57] Iqbal SI, Helge JW, Heitmann BL (2006). Do energy density and dietary fiber influence subsequent 5-year weight changes in adult men and women?. Obesity.

[58] Sammel MD, Grisso JA, Freeman EW, Hollander L, Liu L, Liu S (2003). Weight gain among women in the late reproductive years. Fam Pract.

[59] Ferrara LA, Innelli P, Palmieri V, Limauro S, De Luca G, Ferrara F (2006). Effects of different dietary protein intakes on body composition and vascular reactivity. Eur J Clin Nutr.

[60] Knight EL, Stampfer MJ, Hankinson SE, Spiegelman D, Curhan GC (2003). The impact of protein intake on renal function decline in women with normal renal function or mild renal insufficiency. Ann Intern Med.

[61] Frank H, Graf J, Amann-Gassner U, Bratke R, Daniel H, Heemann U (2009). Effect of short-term high-protein compared with normal-protein diets on renal hemodynamics and associated variables in healthy young men. Am J Clin Nutr.

[62] Friedman AN (2004). High-protein diets: potential effects on the kidney in renal health and disease. Am J Kidney Dis.

[63] Jakobsen LH, Kondrup J, Zellner M, Tetens I, Roth E (2011). Effect of a high protein meat diet on muscle and cognitive functions: a randomised controlled dietary intervention trial in healthy men. Clin Nutr.

[64] Curhan GC, Willett WC, Knight EL, Stampfer MJ (2004). Dietary factors and the risk of incident kidney stones in younger women: Nurses’ Health Study II. Arch Intern Med.

[65] Taylor EN, Stampfer MJ, Curhan GC (2004). Dietary factors and the risk of incident kidney stones in men: new insights after 14 years of follow-up. J Am Soc Nephrol.

[66] de Konig L, Fung TT, Liao X, Chiuve SE, Rimm EB, Willet WC (2011). Low-carbohydrate-diet scores and the risk of type 2 diabetes in men. Am J Clin Nutr.

[67] Halton TL, Liu S, Manson JE, Hu FB (2008). Low-carbohydrate-diet score and the risk of type 2 diabetes in women. Am J Clin Nutr.

[68] Schulze MB, Schulz M, Heidemann C, Schienkiewitz A, Hoffmann K, Boeing H (2008). Carbohydrate intake and incidence of type 2 diabetes in the European Prospective Investigation into Cancer and Nutrition (EPIC)-Potsdam Study. Br J Nutr.

[69] Sluijs I, Beulens JWJ, van der ADL, Spijkerman AMW, Grobbe DE, van der Schouw YT (2010). Dietary intake of total, animal, and vegetable protein and risk of type 2 diabetes in the European Prospective Investigation into Cancer and Nutrition (EPIC) NL study. Diabetes Care.

[70] Bowden RG, Lanning BA, Doyle EL, Slonaker B, Johnston HM, Scanes G (2007). Systemic glucose level changes with a carbohydrate-restricted and higher protein diet combined with exercise. J Am Coll Health.

[71] Gaine PC, Viesselman CT, Pikovsky MA, Martin WF, Armstrong LE, Pescatello LS (2005). Aerobic exercise training decreases leucine oxidation at rest in healthy adults. J Nutr.

[72] Hartman JW, Moore DR, Phillips SM (2006). Resistance training reduces whole-body protein turnover and improves net protein retention in untrained young males. Appl Physiol Nutr Metab.

[73] Thalacker-Mercer AE, Petrella JK, Bamman MM (2009). Does habitual dietary intake influence myufiber hypertrophy in response to resistance training? A cluster analysis. Appl Physiol Nutr Metab.

[74] FAO/WHO/UNU (1985). Energy and protein requirements. Report of a joint FAO/WHO/UNU expert consultation. WHO Technical Report Series, No. 724.

[75] Appel LJ, Moore TJ, Obarzanek E, Vollmer WM, Svetkey LP, Sacks FM (1997). A clinical trial of the effects of dietary patterns on blood pressure. N Engl J Med.

[76] Thorpe P, Evans EM (2011). Dietary protein and bone health: harmonizing conflicting theories. Nutr Rev.

[77] Summerbell CD, Douthwaite W, Whittaker V, Ells LJ, Hillier F, Smith S (2009). The association between diet and physical activity and subsequent excess weight gain and obesity assessed at 5 years of age or older: a systematic review of the epidemiological evidence. Int J Obes.

[78] Bankir L, Bouby N, Trinh-Trang-Tan MM, Ahloulay M, Promeneur D (1996). Direct and indirect cost of urea excretion. Kidney Int.

[79] Taal MW, Brenner BM (2008). Renal risk scores: progress and prospects. Kidney Int.

[80] Bosch JP, Lew S, Glabman S, Lauer A (1986). Renal hemodynamic changes in humans. Response to protein loading in normal and diseased kidneys. Am J Med.

[81] Rodriguez NR, Di Marco NM, Langley S, American Dietetic Association, Dietitians of Canada, American College of Sports Medicine (2009). American College of Sports Medicine position stand. Nutrition and athletic performance. Med Sci Sports Exerc.

[82] Black AE (2000). Critical evaluation of energy intake using the Goldberg cut-off for energy intake: basal metabolic rate. A practical guide to its calculation, use and limitations. Int J Obes.

[83] Gibson RS (2005). Principles of nutritional assessment.

[84] Beasley JM, LaCroix AZ, Neuhouser ML, Huang Y, Tinker L, Woods N (2010). Protein intake and incident frailty in the Women's Health Initiative observational study. J Am Geriatr Soc.

